# The Role of Meteorite Impacts in the Origin of Life

**DOI:** 10.1089/ast.2019.2203

**Published:** 2020-09-15

**Authors:** G.R. Osinski, C.S. Cockell, A. Pontefract, H.M. Sapers

**Affiliations:** ^1^ Institute for Earth and Space Exploration, University of Western Ontario, London, Canada.; ^2^ Department of Earth Sciences, University of Western Ontario, London, Canada.; ^3^ UK Centre for Astrobiology, School of Physics and Astronomy, University of Edinburgh, Edinburgh, UK.; ^4^ Department of Biology, Georgetown University, Washington, DC, USA.; ^5^ Division of Geological and Planetary Sciences, California Institute of Technology, Pasadena, California, USA.; ^6^ Department of Earth Sciences, University of Southern California, Los Angeles, California, USA.

**Keywords:** Origin of life, Impact craters, Hadean environment, Hydrothermal systems, Crater lakes, Lithophytic habitats, Geobiology

## Abstract

The conditions, timing, and setting for the origin of life on Earth and whether life exists elsewhere in our solar system and beyond represent some of the most fundamental scientific questions of our time. Although the bombardment of planets and satellites by asteroids and comets has long been viewed as a destructive process that would have presented a barrier to the emergence of life and frustrated or extinguished life, we provide a comprehensive synthesis of data and observations on the beneficial role of impacts in a wide range of prebiotic and biological processes. In the context of previously proposed environments for the origin of life on Earth, we discuss how meteorite impacts can generate both subaerial and submarine hydrothermal vents, abundant hydrothermal–sedimentary settings, and impact analogues for volcanic pumice rafts and splash pools. Impact events can also deliver and/or generate many of the necessary chemical ingredients for life and catalytic substrates such as clays as well. The role that impact cratering plays in fracturing planetary crusts and its effects on deep subsurface habitats for life are also discussed. In summary, we propose that meteorite impact events are a fundamental geobiological process in planetary evolution that played an important role in the origin of life on Earth. We conclude with the recommendation that impact craters should be considered prime sites in the search for evidence of past life on Mars. Furthermore, unlike other geological processes such as volcanism or plate tectonics, impact cratering is ubiquitous on planetary bodies throughout the Universe and is independent of size, composition, and distance from the host star. Impact events thus provide a mechanism with the potential to generate habitable planets, moons, and asteroids throughout the Solar System and beyond.

## 1. Introduction

When and where life originated on Earth and whether there existed, or exists, life elsewhere in our solar system represent some of the biggest unanswered scientific questions of our time. These questions provide motivation for the near-term robotic exploration of Mars, including the return of samples, as well as the exploration of more distant targets, both within (*e.g.,* Europa and Enceladus) and outside our solar system. But how did life on Earth begin? There is some consensus regarding the requisite conditions for the transition from prebiotic chemistry to living systems: a maintained excess of Gibbs free energy (-Δ*G*), a solvent (water), a mode for the encapsulation and concentration of prebiotic molecules, a mechanism of information storage, and the presence of catalytic molecules such as enzymes (*e.g.,* Pace, 2001; Monnard and Deamer, 2002). Although there is widespread speculation on the geological setting for the origin of life, the earliest geological and biological evidence for early life and the putative conditions on the early Archean or Hadean Earth provide some environmental constraints. Several theories suggest a hot, aqueous environment for the origin of life (*e.g.,* Pace, 1991; Stetter, 2005), and submarine hydrothermal vents have been widely proposed as candidate environments for prebiotic chemistry (*e.g.,* Orgel, 1998; Nisbet and Sleep, 2001; Copley *et al.,* 2007; Martin *et al.,* 2008; Russell *et al.,* 2013). Indeed, while still debated, many phylogenetic tree reconstructions when using molecular analyses of 16S rRNA combined with metabolic studies suggest a hyperthermophilic last universal common ancestor (LUCA) (Woese *et al.,* 1990; Pace, 1991; Di Giulio, 2001, 2003, 2007; Schwartzman and Lineweaver, 2004; Brack *et al.,* 2010). However, there are suggestions that LUCA may have been a mesophilic organism that had a moderate optimal growth temperature of ∼20–45°C (*e.g.,* Miller and Lazcano, 1995; Groussin *et al.,* 2013). Subaerial alkaline hydrothermal environments have also received increasing attention as environments for life's origins, due to lower overall temperatures and the propensity for repeated wetting-drying cycles (Deamer and Georgiou, 2015). In a recent contribution, Westall *et al.* (2018) hypothesized a related, though distinct, hydrothermal-sedimentary environment for the origin of life in the form of the sedimentary layer between oceanic crust and seawater. Further hypotheses for the environment in which life originated include volcano-hosted splash pools in coastal environments (*e.g.,* Fox and Strasdeit, 2013) and floating pumice rafts on early oceans (Brasier *et al.,* 2011).

Unfortunately, the rock record on Earth offers relatively little insight into the early history of Earth and the inner Solar System in general due to constant crustal recycling and resurfacing events over the past 4.5 billion years. The Moon and other Solar System objects, however, preserve substantial portions of their earliest crust; for example, over 50% of the martian geological record older than 3.5 Ga is preserved (Tanaka *et al.,* 2014). When taken together with theoretical and observational constraints on planet formation, numerical simulations, and studied samples from our closest neighbor, the Moon, a clearer picture emerges of the first few hundred million years of Earth's history. While the details are debated, it is now widely accepted that a cataclysmic impact event at ∼4.5 Ga between the proto-Earth and a Mars-sized object (“Theia”) led to the formation of the Moon (*e.g.,* Canup, 2012; Ćuk and Stewart, 2012). For several million years after this event the surface temperature of Earth would have remained well above the known upper temperature limit for life, rendering it inhospitable. Cratering rates over the subsequent few hundred million years are the topic of ongoing debate. Based originally on the determination of a large concentration of ∼3.9 Ga ages in Apollo samples (*e.g.,* Papanastassiou and Wasserburg, 1971; Tera *et al.,* 1974), the concept of the Late Heavy Bombardment (LHB) was proposed (see Chapman *et al.,* 2007; Norman, 2009, and references therein). The scarcity of pre-3.9 Ga ages in Apollo samples as well as similar impact age distributions in some meteorites has led some to believe that the LHB was the result of a lunar terminal cataclysm (*e.g.,* Cohen *et al.,* 2000; Stöffler and Ryder, 2001; Kring and Cohen, 2002). Others have argued that this concentration of impact ages at ∼3.9 Ga is an artifact that reflects sampling biases (*e.g.,* Hartmann, 2003; Zellner, 2017) or age resetting (Boehnke and Harrison, 2016), which resulted in a monotonic decay rate since ∼4.5 Ga. Regardless of whether or not there was a spike in cratering rates at ∼3.9 Ga, there is general consensus that, for the first half billion years of Solar System history, Earth experienced a higher rate of meteorite impacts relative to modern impact flux (*cf.* Chambers [2004] with Zellner [2017]), which further suggests that this prolonged bombardment continued to ∼3.4 Ga.

Intriguingly, the ∼3.7–3.8 Ga Isua greenstone belt of Greenland, which temporally coincides with the end of this period of higher impact flux, has long been proposed to contain the earliest evidence for life, as indicated by the presence of highly negative δ^13^C values within carbonaceous inclusions found in apatite grains (Mojzsis *et al.,* 1996). This coincidence, in part, led to the hypothesis of the impact frustration of life, whereby the intensity of impact cratering would have either precluded the existence of life prior to ∼3.8 Ga (Maher and Stevenson, 1988; Sleep *et al.,* 1989) or extinguished its presence (Chyba, 1993), perhaps multiple times. Higher impact rates prior to ∼3.8 Ga have also been used to suggest a thermal bottlenecking event that would result in the preferential selection of hyperthermophiles and lead to the observed hyperthermophilic bias in modern evolutionary 16S rRNA phylogenetic tree reconstructions (*e.g.,* Nisbet and Sleep, 2001; Kring and Cohen, 2002). Perhaps unsurprisingly, there has been a general tendency in the past few decades to think of impacts as primarily destructive events that would have endangered, rather than enabled, life on Early Earth, as most recently discussed in the work of Sleep (2018). This view was strengthened by the discovery of the ∼200 km diameter Chicxulub impact structure, Mexico, and its link to the mass extinction event that marks the end of the Cretaceous Period ∼66 million years ago (Schulte *et al.,* 2010). However, if the carbon isotope ratios that were found preserved in metasedimentary rocks in Isua, Greenland (Ohtomo *et al.,* 2014), and Labrador, Canada (Tashiro *et al.,* 2017), are indeed evidence of life, then the origin of life would predate ∼3.8 to ∼3.9 Ga such that there would be sufficient evolution and accumulation of primitive organisms to leave an isotopically light carbon signature in the rock record. This is also supported by the recent discovery of putative fossilized microorganisms in rocks found in Quebec, Canada, hosted deposits interpreted as hydrothermal vent precipitates, and with a proposed age of >3.8 Ga and possibly as old at 4.2 Ga (Dodd *et al.,* 2017). These findings beg the question: Why, or rather how, did life originate during such an inhospitable time in Earth's history—the Hadean—when impact rates were orders of magnitude higher than at the present day?

**FIG. 1.**
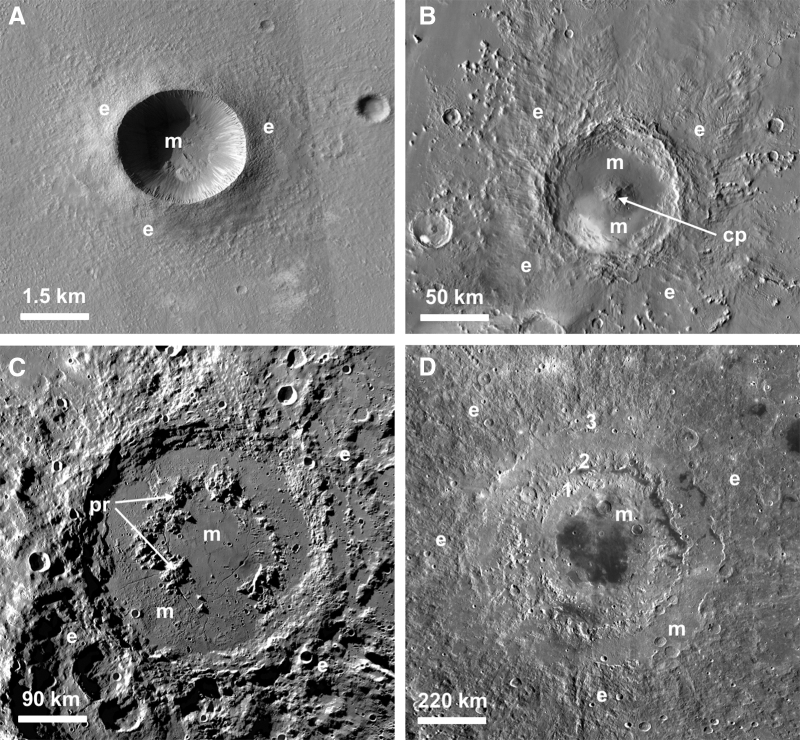
Images of impact craters showing the change in morphology with increasing size. (**A**) The 3 km diameter Zumba Crater on Mars, a prototypical simple crater. Note the impact ejecta deposits (“e”) and the impact melt deposits (“m”) on the crater floor. Portion of Mars Reconnaissance Orbiter High Resolution Imaging Science Experiment (HiRISE) image PSP_003608_1510. NASA/JPL/University of Arizona. (**B**) The 93 km diameter Pettit Crater on Mars, a well-preserved complex crater with a central peak (“cp”). THEMIS day-time mosaic. NASA/JPL/Arizona State University. (**C**) With increasing diameter, central peaks are replaced by peak-rings (“pr”) as in the case of the ∼320 km diameter Schrödinger Crater on the Moon. The crater has well-preserved ejecta deposits, and impact melt occurs both inside and outside the peak-ring. Lunar Reconnaissance Orbiter (LRO) Wide Angle Camera (WAC) mosaic. NASA/JPL/ASU. (**D**) The largest impact features in the Solar System are termed impact basins, such as the ∼900 km diameter Orientale Basin on the Moon shown here. It contains three ring features labeled 1–3. LRO WAC mosaic. NASA/JPL/ASU.

Here, we offer a solution that necessitates a paradigm shift in our view of the biological consequences of meteorite impact events on Earth and elsewhere in the Universe. We argue that impact events are not just isolated catastrophic geological events but a fundamental process in planetary evolution that plays an important role in the origin of life and in controlling planetary habitability. In this contribution, we build on ideas first expressed by earlier workers (*e.g.,* Chyba, 1993; Kring, 2000; Osinski *et al.,* 2001; Cockell, 2006; Sleep, 2018; Schmieder and Kring, 2020) and provide a modern comprehensive treatise of the role of meteorite impacts in the origin and early evolution of life. This work is grounded in our own extensive field and laboratory studies of the impact cratering record on Earth, which now numbers 198 confirmed impact craters (Osinski and Grieve, 2019; see also www.impactearth.com for an up-to-date inventory), and synthesized with results from other studies. We review the beneficial effects for microbial life ranging from generating *conditions* conducive to the origin of life (*e.g.,* clays that can act as catalytic substrates for organic reactions, serpentinization, atmospheric generation of hydrogen cyanide, and hot spring environments) to varied *habitats* for life that persist long after an impact event, including transient hydrothermal systems, endolithic habitats in impact glasses and impact shocked rocks, and impact crater lakes. The purpose of this contribution is not to repudiate or argue against any previously proposed environments for the origin of life, but to synthesize ideas and data on the potential importance of meteorite impact craters. While impact craters represent a standalone environment, we show that they can also provide an alternative mechanism or pathway by which to generate previously proposed prebiotic environments, such as submarine hydrothermal vents, subaerial hydrothermal fields, and even pumice rafts. Furthermore, unlike other geological processes such as volcanism or plate tectonics, impact cratering is ubiquitous on rocky and icy bodies in the Solar System (Fig. 1) and is independent of size, internal heating mechanisms, and distance from the Sun. Impact events thus potentially provide a mechanism to generate habitable planets, satellites, and even asteroids throughout and beyond the Solar System with implications for investigating the habitability of exoplanets.

We conclude this contribution with a discussion of how meteorite impacts would have provided conditions suitable for the emergence of life on Mars. This is particularly important given the ongoing development of a framework for Mars Sample Return and the NASA Perseverance and ESA Rosalind Franklin rover missions planned to launch in 2020 and 2022, respectively. While impact craters have featured prominently in the surface exploration of Mars in the past, they have typically been viewed as sites offering bedrock exposure (*e.g.,* Eagle, Endurance, and Victoria Craters during the Mars Exploration Rover Opportunity's exploration [Squyres *et al.,* 2004]) or as sedimentary basins providing unique climate records (*e.g.,* Gale Crater currently being explored by the Mars Science Laboratory Curiosity rover [Grotzinger *et al.,* 2012] and Jezero Crater, the planned landing site for NASA's 2020 Perseverance rover mission [Goudge *et al.,* 2018]). We hope that this contribution highlights the importance of martian impact craters as prime astrobiological targets, not just for their post-impact sedimentary record but also as primary potential habitats for life.

## 2. Impact Cratering: A Planetary Geobiological Process

Impact cratering has generally been considered from the perspective of geology rather than biology. Over the past few decades it has become clear that the formation of a meteorite impact crater is a unique geological event; the concentrated nature of the energy release at a single point on a planetary surface, the virtually instantaneous nature of the process (*e.g.,* seconds to minutes), and the high strain rates involved (∼10^4^ s^−1^ to 10^6^ s^−1^) set the impact cratering process apart from any other geological process, such as volcanism and tectonism (for comprehensive overviews, see Melosh [1989] and Osinski and Pierazzo [2012]). From a geological standpoint, the formation of an impact crater has historically been divided into three main stages, originally defined in the work of Gault *et al.* (1968): (1) contact and compression, (2) excavation, and (3) modification. Cockell and Lee (2002) suggested that these geological changes could be used to define different phases of biological recovery within an impact crater as follows: (1) phase of thermal biology resulting from the thermal anomaly of impact, (2) phase of impact succession and climax as the crater geology and surface expression change over time, and (3) phase of ecological assimilation. We revisit these previous approaches and distinguish four distinct phases in the origin and subsequent evolution of an impact crater. The important attributes of the various impact craters mentioned in this contribution are provided in Table 1.

**Table 1.** Main Attributes of Craters Mentioned in the Text

Crater	Location	Coordinates	Diameter (km)	Age (Ma)
Bosumtwi	Ghana	6° 30' 18” N; 1° 24' 35” W	10.5	1.03 ± 0.02
Chesapeake Bay	USA	37° 17' 56” N; 76° 2' 14” W	90	35.5 ± 0.6
Chicxulub	Mexico	21° 18' 42” N; 89° 25' 53” W	200	64.98 ± 0.05
East Clearwater	Canada	56° 3' 37” N; 74° 7' 10” W	26	460–470
El'gygytgyn	Russia	67° 29' 24” N; 172° 4' 15” E	18	3.58 ± 0.04
Haughton	Canada	75° 22' 39” N; 89° 39' 13” W	23	23.5 ± 2
Kärdla	Estonia	58° 58' 33” N; 22° 46' 44” E	4	∼455
Lappajärvi	Finland	63° 8' 11” N; 23° 39' 41” E	23	77.85 ± 0.78
Ries	Germany	48° 52' 37” N; 10° 33' 21” E	24	15.1 ± 0.1
New Quebec	Canada	61° 16' 37” N; 73° 39' 38” W	3.4	1.4 ± 0.1
Sudbury	Canada	46° 35' 36” N; 81° 8' 20” W	250	1850 ± 3
West Clearwater	Canada	56° 12' 45” N; 74° 31' 39” W	36	286 ± 2.2

All attributes of terrestrial craters are from www.impactearth.com

### 2.1. Thermobaric phase

**FIG. 2.**
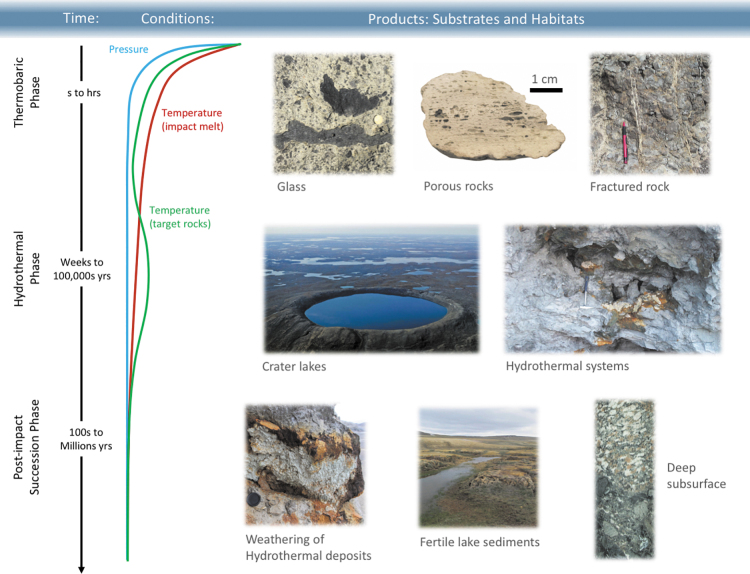
Variation in pressures and temperatures from the thermobaric to equilibrium phase of crater formation and the major substrates and habitats associated and utilized during each stage. Note that a habitat or substrate may remain used during subsequent stages (*e.g.,* porous rocks and glasses can remain important habitats during the equilibrium phase). The temperature curves represent an average and will be different for different parts of a crater (see text for details).

The thermobaric phase is the initial high-pressure and high-temperature phase during which an impact crater is formed (Fig. 2) and can be broadly described and subdivided into the three main stages as originally defined in the work of Gault *et al.* (1968). This work is concerned with *hypervelocity* impact craters that form when the asteroidal or cometary projectile is large enough to pass through the atmosphere with little or no deceleration and impact occurs at or near its original cosmic velocity (French, 1998). Such hypervelocity impacts produce shock waves in the target, whereas smaller projectiles lose most of their original kinetic energy in the atmosphere and produce small, meter-sized “penetration craters,” leaving much of the projectile intact as meteorites.

**FIG. 3.**
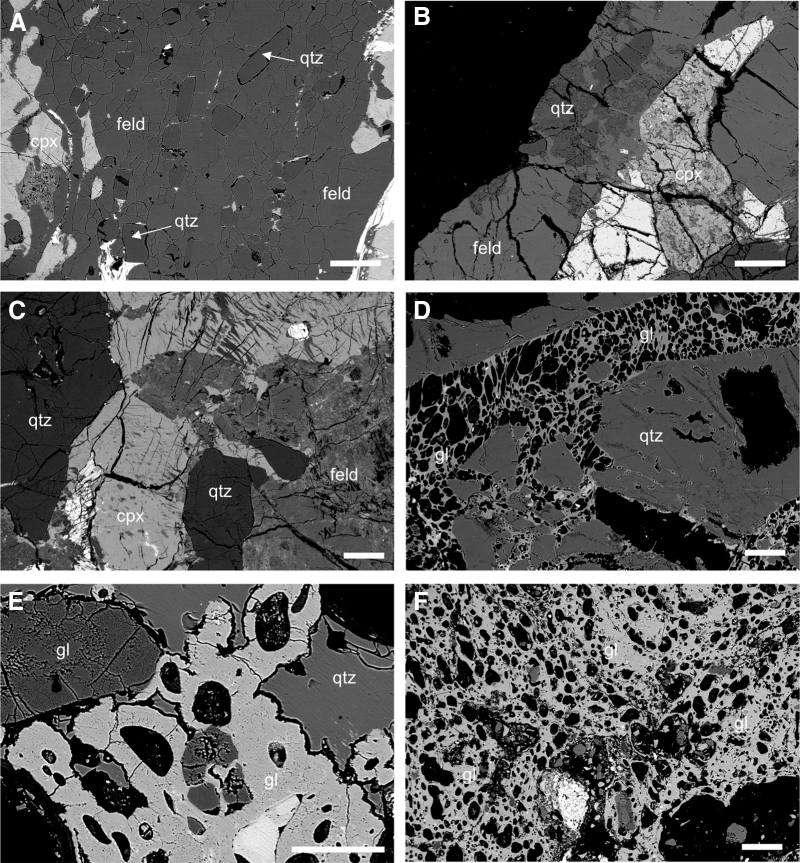
Backscattered electron images showing the progressive physical changes in crystalline rocks from the Haughton impact structure with increasing shock pressure. (**A**) Unshocked gneiss with essentially no porosity and few fractures. (**B**) In Shock Level 2 samples (∼5–10 GPa), all minerals are fractured to varying degrees. Average porosity is ∼1%. (**C**) Shock Level 3 (∼10–30 GPa). All minerals are heavily fractured with planar deformation features in quartz and average porosities of ∼2–5%. (**D**) In Shock Level 5 (∼35–55 GPa) samples there is extensive development of diaplectic glass in quartz. Feldspar has undergone melting to form vesiculated glass. Average porosities are ∼18%. (**E**) Shock Level 6 (∼55–60 GPa). All minerals are transformed to diaplectic glass or partially to completely melted and transformed to mineral glasses. Average porosities are ∼44% (**F**) In Shock Level 7 (>60 GPa) samples, all minerals have melted and transformed to glass. Average porosities are ∼63%. All scale bars are 100 μm. Abbreviations: qtz = quartz; feld = feldspar; cpx = clinopyroxene; gl = glass. The black regions in all images are holes in the thin sections. Porosity values are from Pontefract *et al.* (2014).

**FIG. 4.**
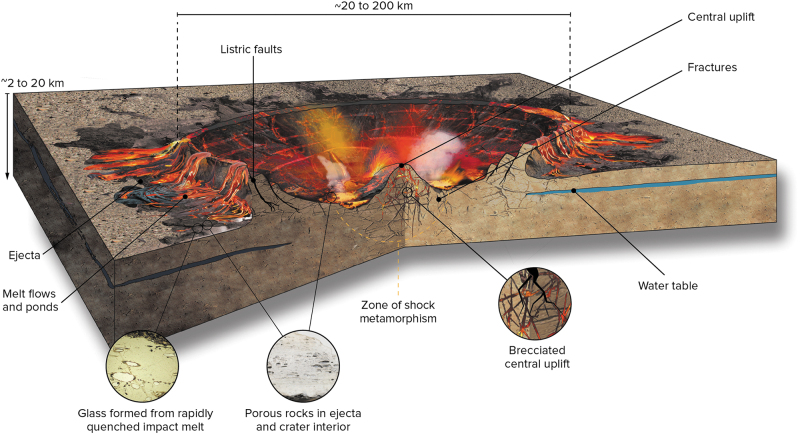
Artistic rendition of a typical complex impact crater at end of thermobaric phase. At this time, the hydrosphere remains severely disrupted, and the crater interior is filled with impact melt deposits that are superheated to temperatures over 2300°C. Ponds and flows of hot impact melt also occur in patches on top of the ballistic impact ejecta deposits in the crater exterior. The target rocks inside the crater are heavily fractured and shock metamorphosed with shock pressures increasing toward the center.

The initial contact and compression stage begins when the projectile, either an asteroid or comet, contacts the surface of the target (Melosh, 1989). The pressures at the point of impact are typically several hundred gigapascals (Fig. 2). During this brief stage, which lasts no more than a few seconds for even the largest event (Fig. 2), the kinetic energy of the projectile is partitioned into kinetic energy and transferred to the target. The resulting motion of the target rocks leads to the formation of a crater, and the dissipation of the internal energy leads to progressive shock metamorphism of target rocks that produce a series of “shock effects” (French and Koeberl, 2010). These shock effects range from fractures and shatter cones at low shock pressures through to the formation of planar deformation features and high-pressure polymorphs, and eventually the generation of diaplectic or solid-state glasses (Figs. 2–4). Importantly, with increasing levels of shock (*i.e.,* increasing pressure), there is a notable increase in porosity and permeability in target rocks (Singleton *et al.,* 2011) (Fig. 2) creating important new lithophytic (rock-based) microbial habitats (see Section 4.2). Closer to the point of impact, the melting of a large volume of target rock occurs upon decompression from high shock pressures and temperatures (Osinski *et al.,* 2018) (Figs. 2, 4).

The crater itself begins to form during the subsequent excavation stage when interactions between outward-directed shock waves and the downward-directed rarefaction waves generate the “transient cavity.” For relatively small impact events (<2–4 km on Earth, ∼10 km on Mars, and up to ∼20 km diameter on the Moon), the transient cavity undergoes only minor modification, which results in the formation of a bowl-shaped simple crater (Fig. 1A) lined with a mix of melted, shock metamorphosed, and fractured material (collectively termed crater-fill deposits), surrounded by impact ejecta deposits. The final products of impact are a wide variety of “impactites” (rocks affected by impact metamorphism) (Stöffler and Grieve, 2007), ranging from melt-free to glass-bearing breccias to large kilometer-thick coherent sheets of igneous-textured impact melt rocks (Osinski *et al.,* 2018).

For larger impact events, the transient cavity is gravitationally unstable, and a complex impact crater is formed (Kenkmann *et al.,* 2012). Such craters comprise a faulted crater rim, a central peak emergent through a lens of crater-fill deposits (impact melt rock and/or glass-bearing breccias) (Figs. 1B, 4), and impact ejecta deposits. At larger diameters, the central peak is replaced by a peak ring (Fig. 1C) and, eventually, a series of rings in multi-ring basins (Fig. 1D), the largest impact features in the Solar System. A phenomenon unique to complex craters and basins is that, during the modification stage, crustal and even mantle rocks are instantaneously brought to the surface from depth in the form of central uplifts (Figs. 1B–D, 4). On Earth, plate tectonics is the main process responsible for crustal recycling and for transporting deep crustal and mantle materials to the surface or near subsurface (Condie, 2016). In the absence of plate tectonics (*i.e.,* for most objects in the Universe), impact cratering will thus be the primary agent of resurfacing, and impact ejecta deposits and central uplifts will be the primary mechanism to exhume ultramafic rocks, such as peridotite and basalt, from the deep subsurface. Furthermore, it is thought that plate tectonic rates during the Hadean were slower than the present-day and that the Hadean oceanic crust was much thicker (*e.g.,* Korenaga, 2012); thus, even in the presence of plate tectonics, impacts may have played a major role in exhuming deep crustal and mantle rocks. The amount of exhumation or structural uplift (SU) in the central uplifts of complex craters increases with increasing crater diameter (D), according to the relationship SU = 0.06D^1.1^ (Grieve *et al.,* 1981).

Importantly, in addition to exhuming ultramafic rocks and their alteration products on early Earth, impact events also excavate, mix, and disperse a diverse assemblage of unshocked and shocked target rocks and impact melt products over large portions of Earth's surface through the emplacement of ejecta deposits; material is distributed up to 5 crater radii in proximal ejecta deposits (Figs. 1, 4) and, during larger kilometer-sized events, may be transported hundreds to thousands of kilometers in distal ejecta deposits (Osinski *et al.,* 2012). There are few reliable estimates for the depth of excavation of impact ejecta deposits. For simple craters, observations from Barringer Crater suggest a value of ∼0.08 D (Shoemaker, 1960) for the continuous ejecta blanket. For complex craters, the depth of excavation has been estimated at ∼0.05 D for the continuous ejecta blanket and 0.12 D for the overlying upper layer of ejecta found in many craters of this type (Osinski *et al.,* 2011).

While the pressures and temperatures during the thermobaric phase are indeed “extreme” by geological standards, these conditions are confined to a very small portion (approximately half the diameter) of the original transient cavity and to an even smaller proportion of the final crater. Indeed, it has been shown that even within rocks that were highly shocked and encased in impact melt rocks, bioessential elements (Pontefract *et al.,* 2012) and even extant bacteria (Hazael *et al.,* 2017) can escape volitization and survive the impact event. On a planetary scale, numerical modelling has also shown that there is no plausible scenario in which the habitable zone could be fully sterilized during the period of intense cratering on early Earth (Abramov and Mojzsis, 2009; Grimm and Marchi, 2018). On the contrary, it is during the thermobaric phase that the first new habitats are created through fracturing, shock metamorphism, and melting (see Section 4) (Figs. 2–4).

### 2.2. Hydrothermal phase

**FIG. 5.**
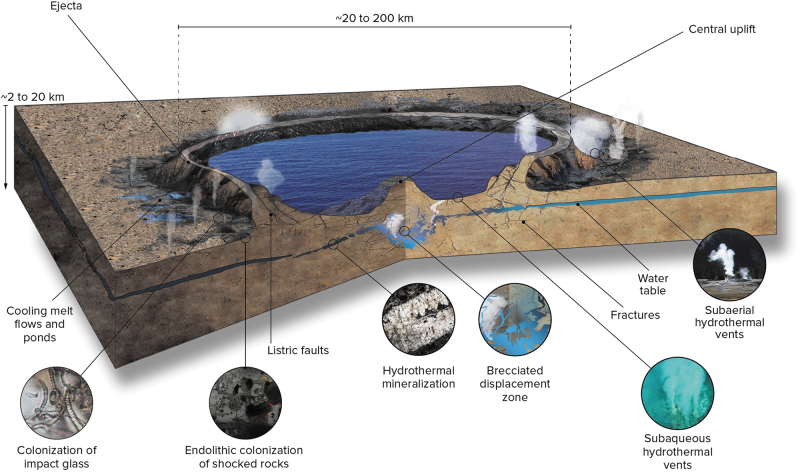
Artistic rendition of a typical complex impact crater during the hydrothermal phase. If conditions permit, a hydrothermal system and crater lake can develop, along with several other habitats (described in Section 4).

At the end of thermobaric phases, the biosphere is significantly disrupted in the immediate area surrounding impact with local areas of sterilization. Subsequently, primary succession of the landscape will occur (Cockell and Lee, 2002). One of the significant outcomes of the thermobaric phase is the disruption of the local hydrosphere and/or cryosphere. Over the subsequent days to years, groundwater, if present, will flow into the newly formed hydraulic void represented by the crater structure. Coupled with precipitation and the melting of surface and subsurface ice deposits, where applicable, the influx of water into the crater depression can lead to the generation of a crater lake, potentially within days or weeks (Figs. 2, 5). Crater lakes in and of themselves represent important habitats and are discussed in Section 4.3.

It has become increasingly clear from studies of craters on Earth over the past couple of decades that interaction of fluids from crater lakes and other sources with hot impact-generated and impact-altered rocks can generate transient hydrothermal systems (*e.g.,* Newsom, 1980; Naumov, 2005; Kirsimäe and Osinski, 2012; Osinski *et al.,* 2013) that can persist for tens of thousands of years. Cockell and Lee (2002) included hydrothermal systems in the phase of thermal biology since hydrothermal systems are the result of the thermal anomaly caused by the impact event (see below). However, as these systems merit special attention on account of their ubiquity and interest to astrobiology, we discuss impact-generated hydrothermal activity as a separate phase.

There are three main sources of heat for impact-generated hydrothermal systems (Kirsimäe and Osinski, 2012; Osinski *et al.,* 2005a, 2013): (1) impact melt rocks and impact melt-bearing breccias; (2) elevated geothermal gradients in central uplifts; and (3) energy deposited in central uplifts due to the passage of the shock wave. In general, impact melt-bearing lithologies are the major driver of hydrothermal systems in small to midsized impact craters, whereas in larger craters the heat input from the uplifted geothermal gradient in central uplifts increases. As noted in Section 2.1 and shown in Fig. 1B–1D, complex craters are defined by the presence of a central uplift that, in general, brings rocks to the surface from a depth approximately equal to one-tenth of the diameter: for a 100 km diameter impact crater on Earth, the central uplift rocks would be derived from ∼10 km depth.

Whereas the initial thermobaric phase lasts only a few minutes, modelling and dating studies suggest that the heat-driven effects of the impact event persist over timescales of significance to microbial colonization and evolution. Modelling of impact-generated hydrothermal systems and detailed geological studies of hydrothermal deposits suggest that they span the spatial extent of the impact structure and rapidly cool to temperatures consistent with the growth of hyperthermophilic and thermophilic microorganisms (Zürcher and Kring, 2004; Abramov and Kring, 2007). The exact duration of impact-generated hydrothermal systems remains a topic of ongoing study and debate. Early studies of small to midsized craters, such as the 4 km diameter Kärdla and 23 km diameter Haughton structures, suggested that such systems would last thousands to tens of thousands of years (Jõeleht *et al.,* 2005; Parnell *et al.,* 2005). Cooling in these small to midsized structures is relatively rapid due to mainly convective heat transport (Kirsimäe and Osinski, 2012). However, recent high-precision age dating of the 23 km diameter Lappajärvi impact structure, Finland, suggests that post-impact cooling and hydrothermal circulation may have persisted for over 1 million years (Schmieder and Jourdan, 2013; Kenny *et al.,* 2019). For larger craters, numerical modelling and age dating of the hydrothermal systems in the ∼250 km diameter Sudbury and ∼200 km diameter Chicxulub structures yield durations on the order of 1 to 2 million years (Ames *et al.,* 1998; Abramov and Kring, 2004, 2007). As emphasized by Kirsimäe and Osinski (2012), the longer cooling times in large structures can be explained by the depth partitioning of cooling mechanisms/regimes. In these large structures, the more effective convective heat transport takes place only near the surface, whereas in deeper parts of the crater conduction is the dominant form of heat transport. Importantly, for midsized to large structures, meteoric and groundwater may be tapped, penetrating down several kilometers along faults and fractures, warming, and then circulating back, delivering additional heat to the near surface and thereby prolonging hydrothermal fluid flow (Abramov and Kring, 2004; Kirsimäe and Osinski, 2012).

It is important to note that impacts into planetary crusts result in massive fracturing and brecciation of the substrate as well as disruption of the deep subsurface (Figs. 4, 5), generating a large volume of reactive surface area. For example, a ∼200 km diameter crater potentially harbors a subsurface habitable volume of ∼50,000 km^3^ (Abramov and Kring, 2004). This fracturing is analogous to the seafloor fracturing and subsequent generation of energy disequilibrium proposed in the work of Russell *et al.* (2013). To the knowledge of the authors, nobody has yet attempted to model the duration of hydrothermal systems associated with the many >1000 km diameter impact basins that would have formed during the first half billion years of Earth's history.

The end result of the hydrothermal phase is a large volume of variably altered pre-impact target rocks and impactites. Hydrothermal mineral assemblages in a particular impact crater are governed by local physicochemical conditions, largely controlled by the type (or types) of target rock and fluid sources (*e.g.,* meteoric vs. seawater) involved. The three dominant groups of alteration minerals in terrestrial craters are clays, zeolites, and carbonates (Naumov, 2002; Osinski *et al.,* 2013). However, as noted in the previous section, during the thermobaric phase of crater formation, rocks from tens of kilometers' depth will be exhumed during the formation of 100–1000 km diameter craters that would have been common during the Hadean. We have no preserved craters dating from this time, but it seems expected that ultramafic rocks from the deep subsurface would have been exhumed from depth and brought to the surface or near subsurface. This is important, as the hydrothermal alteration product of such ultramafic rocks, serpentine, has been proposed to have potentially played a role in the emergence of life, both through the release of hydrogen—which is an energy source for metabolism—via the serpentinization process and due to its ability to participate in the abiotic formation of organic compounds (*e.g.,* Schulte *et al.,* 2006; Sherwood Lollar *et al.,* 2007; Russell, 2007; Müntener, 2010; Russell *et al.,* 2010; Sleep *et al.,* 2011).

Another factor that amplifies hydrothermal alteration in impact craters is the presence of glasses and highly shocked materials that are more susceptible to alteration than their unshocked equivalents. Importantly, these transient hydrothermal systems can provide the substrates (see Section 3) and habitats (see Section 4) necessary to support the origins and early evolution of life.

### 2.3. Post-impact succession phase

Once the impact-generated heat has fully dissipated from a crater and hydrothermal circulation has ceased, ambient surface temperatures return to pre-impact values: we consider this as the beginning of the next and longest-lived phase of post-impact ecological succession (Fig. 2) that will proceed until an ecologically stable environment is reached. In this phase, provided they are not eroded, impact-altered rocks will persist, and their impact-generated physical and chemical properties will continue long after impact. The Haughton impact structure in the Canadian High Arctic offers a unique opportunity to investigate the effects of the original impact event on the process of ecological succession, where the present-day polar-desert conditions of the High Arctic limit weathering, resulting in impact rocks that are relatively well preserved (Osinski *et al.,* 2005b). The lack of vegetation and extensive soils in this environment means that the physical and chemical characteristics of the impactites dominate the behavior and distribution of surface microbial communities (Cockell *et al.,* 2005). Thus, the effects of impact metamorphism are clearly seen on the present-day microbial communities and have likely influenced the distribution of microbial biomass in the crater environment since impact. Indeed, as outlined in Section 4.2., the continuing influence of impact-altered porosity in metamorphic and sedimentary rocks within the Haughton impact structure and their suitability as microbial habitats ∼23 million years after impact (Cockell *et al.,* 2002; Cockell and Osinski, 2007; Pontefract *et al.,* 2014, 2016) illustrates the longevity of this phase.

After the cessation of hydrothermal activity, crater lakes may persist, albeit periodically, throughout geological time, providing relatively stable habitats that can persist for as long as the topographic expression of the crater is retained. On Earth today, lakes in craters such as Manicouagan and West and East Clearwater attest to the potential for these features to persist over hundreds of millions of years, although the biota that inhabit the lakes will experience turnover and change in diversity as climatic conditions within which the crater resides change over time. On Devon Island, the post-impact crater biosphere has been significantly influenced by the presence of post-impact crater lake sediments, known as the Haughton Formation. These deposits not only represent the only record of the Miocene period in the Canadian Arctic (Hickey *et al.,* 1988; Osinski and Lee, 2005; Rybczynski *et al.,* 2009) but also are the most fertile substrate for tens of kilometers around the Haughton structure (Cockell *et al.,* 2001), a modern-day expression of the concentration of nutrients in a crater lake environment (Fig. 2). The morphology of the crater itself has also provided unique habitats for macrofauna: Cockell *et al.* (2003) demonstrated how the fracturing of rocks by the impact event at the Haughton impact structure has created habitats for birds and other modern avifauna and micron-scale refugia for cyanobacteria.

### 2.4. Ecological assimilation phase

Eventually, a crater may be eroded, buried, and/or subducted such that the surface ecology, geology, and topology at the original site of impact are indistinguishable from the outlying areas (at least with respect to the effects of impact). On Earth, this process is significant, and it is primarily driven by plate tectonics and wind/hydrological erosion. Although atmospheric density leads to differences in impact dynamics on Earth and Mars, the characterization of 198 craters on Earth (Osinski and Grieve, 2019) compared to the greater than 600,000 craters greater than 1 km diameter on Mars (Robbins and Hynek, 2012) is testament to the extent to which these features are attenuated over geological time on Earth. Nevertheless, the craters that are known on Earth can influence their local ecologies for millions of years and potentially up to billions of years. Indeed, often ecological anomalies provide the first indication of the presence of impact structures (Cockell and Lee, 2002). Thus, although many impact craters are eventually destroyed, it is clear that even on the geologically dynamic Earth, these structures can influence their associated biota over planetary lifetimes.

## 3. The Role of Impacts in Providing the Building Blocks of Life

Life as we know it is based on a set of bioessential elements fundamental to known organic compounds, including hydrogen, oxygen, nitrogen, phosphorus, and sulfur, based on a carbon backbone (*e.g.,* Ehrenfreund *et al.,* 2002). While the obligatory inclusion of these elements in putative extraterrestrial life can be debated, the prevalence of carbon in the Solar System, as well as the stable nature of its chemical bonds, suggests that all life would be preferentially carbon-based (*e.g.,* Rothschild and Des Marais, 1989; Zeki *et al.,* 1999; Rothschild, 2008). In this section, we show how impacts can deliver and/or generate many of the chemical ingredients required for life. We also note, but do not discuss here, the idea that large impacts may have played a role in delivering N_2_, CO_2_, and other gasses, that may have provided much of the greenhouse warming needed to offset the faint young Sun, thereby improving the overall habitability of Hadean Earth. Essentially, the hypothesis is that volatile-rich asteroids and comets would have released a large fraction of their volatiles directly into the atmosphere when they impacted Earth (*e.g.,* see Kasting, 1990, 2004).

### 3.1. Delivering the chemical ingredients for life

It has long been suggested that during the early history of Earth, asteroids and comets may have been responsible for the delivery of intact organic molecules and volatiles to early Earth (*e.g.,* Chyba, 1990; Delsemme, 1991; Chyba and Sagan, 1992; Brack and Pillinger, 1998). Chyba and Sagan (1992) proposed that the four main extraterrestrial sources for organic molecules on Earth are interplanetary dust particles, airbursts, meteorites, and cometary impacts. Interplanetary dust particles (IDPS), also known as micrometeorites, are the dominant present-day source of extraterrestrial organic matter to Earth. These ∼5–500 μm sized particles contain an average of ∼12 wt % C (Thomas *et al.,* 1993) and comprise a range of organic material, including aromatic and aliphatic compounds, as well as other mineral phases (*e.g.,* Clemett *et al.,* 1993; Keller *et al.,* 2004; Matrajt *et al.,* 2012). Matrajt *et al.* (2006) conducted experiments suggesting that a few percent of organic molecules within micrometeorites would survive atmospheric entry. Maurette (1998) provided a comprehensive review of the potential role and contribution of carbonaceous micrometeorites to the synthesis of prebiotic molecules on early Earth. Airbursts are a specific category of impacts where the projectile explodes at some height in the atmosphere but leaves no crater. Relatively little work has been done on the survival of organics during airburst events; however, it seems that both IDPs and airbursts are a likely source of intact organics on Hadean Earth (*e.g.,* Chyba, 1990; Brack and Pillinger, 1998; Maurette, 1998).

The third important extraterrestrial source for organics is meteorites (*i.e.,* objects that are large enough to survive passage through Earth's atmosphere but small enough to not form hypervelocity impact craters). Meteorites can be classified into three main groups (Lauretta and McSween, 2006): stony—with two major subgroups, chondrites and achondrites—iron, and stony-iron meteorites. Carbonaceous chondrites are one of the three major classes of chondrites and, as their name implies, contain variable amounts of organic molecules. They comprise ∼5% of meteorite falls. The organic inventory of the Murchison meteorite, a CM2 carbonaceous chondrite that fell in Australia in 1969, represents the most thoroughly studied object of this type due to the available mass (∼100 kg) and minor terrestrial contamination. Over a dozen amino acids have been detected in Murchison, including glycine, alanine, and glutamic acid (*e.g.,* Kvenvolden *et al.,* 1970; Pizzarello and Cronin, 1998; Pizzarello *et al.,* 2003). If we consider all meteorite classes, a total of approximately 80 amino acids have been reported (see Burton *et al.,* 2012, for a review). In a recent study, Furukawa *et al.* (2019) provided the most conclusive evidence to date for the presence of extraterrestrial ribose and other bioessential sugars in Murchison and two other carbonaceous chondrites. The availability of a large amount of carbonaceous chondrite material has resulted in a large number of origin-of-life experiments. For example, when subjected to conditions simulating a hydrothermal vent, the insoluble material in the Murray meteorite released aromatic and heteroaromatic hydrocarbons including alkyl dicarboxylic acids up to C18 in chain length (Yabuta *et al.,* 2007).

Another group of meteorites pertinent to the geobiological perspective are iron meteorites, representing ∼6% of meteorite falls (Lauretta and McSween, 2006). Iron meteorites are comprised of >95% Fe and Ni. As discussed by Sleep (2018), Fe and Ni metal are out of equilibrium with water and on Hadean Earth would have reacted to generate hydrogen and produced reducing microenvironments. It has also been shown that iron meteorites are readily used by iron-oxidizing bacteria as a source of energy (González-Toril *et al.,* 2005).

By their very existence, the presence of meteorites means that the organic materials contained within them survived passage through Earth's atmosphere and the low-energy impact with Earth's surface intact, with the exception being the outer few millimeters that is melted to form a fusion crust (Scott, 2011). In relatively small impacts producing simple craters up to ∼2 km in diameter, it is also well known that fragments of the projectile survive largely intact as meteorites and are distributed within the host crater and around it in ejecta deposits (*e.g.,* Barringer Crater in Arizona; Nininger, 1956).

To date, of all known Solar System objects, comets have the largest organic inventory and likely contributed substantially to the organic inventory of early Earth (Ehrenfreund and Charnley, 2000; Despois and Cottin, 2005). Various remote observations and *in situ* missions over the past several years have built up a more complete picture of comet compositions. Early studies of Comet Halley in 1986 found that dust particles from the nucleus were ∼14% organic carbon by mass (Ehrenfreund and Charnley, 2000). Later studies of comets Hyakutake (1996) and Hale-Bopp (1997) revealed the presence of over two dozen biologically relevant organic molecules including ammonia, methane, acetylene, acetonitrile, hydrogen isocyanide, formic acid, isocyanic acid, cyanoacetylene, formamide, and thioformaldehyde (*e.g.,* Biver *et al.,* 1997; Lis *et al.,* 1997). More recently, the Rosetta spacecraft performed the most detailed study of a comet to date (comet 67P/Churyumov-Gerasimenko). Phosphorus and a rich inventory of organic molecules, including methyl cyanate, acetone, propionaldehyde, and acetamide, were detected on the surface (Goesmann *et al.,* 2015) and glycine, methylamine, and ethylamine were detected in the coma (Altwegg *et al.,* 2016). But surely such organic phases would not survive hypervelocity impacts?

Several workers have conducted experiments to investigate the survivability of organics and other bioessential materials during impact. In short, a large number of experiments conducted over a range of shock pressures up to ∼40 GPa have shown that a large fraction of amino acids and organic molecule biomarkers can survive hypervelocity impact (*e.g.,* Blank *et al.,* 2001; Bowden *et al.,* 2009; Parnell *et al.,* 2010a). Scaling up energy several orders of magnitude, in a series of numerical modelling studies Pierazzo and Chyba (1999, 2002) indicated that amino acids would survive the shock heating during the impact of kilometer-sized comets.

In addition to organics, comets have also been shown to contain substantial amounts of the light elements important for life: C, H, O, and N dominate 30% of cometary grains (Irvine *et al.,* 2000). As we noted at the beginning of this section, large impacts would have delivered N_2_, CO_2_, and other gasses, that may have contributed to the greenhouse warming needed to offset the faint young Sun, thereby improving the overall habitability of Hadean Earth. Of relevance here is that Kasting (1990) proposed that much of the carbon delivered by cometary impacts may have been released initially as CO, rather than CO_2_. This is important as it has been proposed that CO was an important trace gas on prebiotic Earth because of its high free energy and its ability to catalyze important reactions involved in prebiotic synthesis (Kasting, 2014).

### 3.2. Generating the chemical and mineral ingredients for life

In addition to comets and asteroids delivering intact molecules, Chyba and Sagan (1992) proposed that organics could also be derived from atmospheric heating due to fast-expanding vapor plumes and via the post-impact recombination of simple organic components. Several studies have shown that amino acids can be synthesized due to atmospheric shock heating in primitive atmospheres (*e.g.,* Bar-Nun *et al.,* 1970; Bar-Nun and Shaviv, 1975). Cyanides are considered to be among the most important compounds for the generation of prebiotic molecules (*e.g.,* Ferris and Hagan, 1984; Orgel, 2004): studies have shown that concentrated solutions of hydrogen cyanide (HCN) can form nucleic acid bases and that mixtures of HCN with carbon compounds can produce amino acids (Ferris and Hagan, 1984). Work by Miller (1953) and Zahnle (1986) used lightning and ultraviolet radiation to drive the chemical generation of HCN; however, these products are quickly destroyed and were not able to be effectively concentrated. Kurosawa *et al.* (2013) showed that HCN formation in meteorite impacts during the LHB could have provided an excellent mechanism for the production and concentration of cyanides, a hypothesis recently supported by the numerical modeling of Devon *et al.* (2018). A portion of the organic molecules delivered from meteorites and cometary material thermally decomposes in the atmosphere and, reacting with the atmospheric N_2_, can then form CN radicals. Kurosawa *et al.* (2013) showed that after a small impact (<1 km in diameter) the density of HCN created is approximately 10 mol/m^2^ over a 100 km^2^ surface area, equivalent to between 500 and 10,000 years of HCN generation via lighting.

During the Hadean, when impact events of this size and larger were frequent, the amount of HCN generated through impacts would have been significant (Devon *et al.,* 2018) and could have been concentrated to a level allowing for the synthesis of some amino acids (Patel *et al.,* 2015). Importantly, Patel *et al.* (2015) also showed that HCN was an important precursor, not only to amino acid synthesis but to the synthesis of RNA, wherein the production of atmospheric HCN could result in the generation of ferrocyanide and phosphate salts, which then rain down on the surface and are concentrated through evaporation. This evaporative process, along with the presence of ultraviolet light, is a necessary step in the chemical pathway leading to the abiotic synthesis of ribonucleotides, and thus far remains the only known prebiotic formation of ribonucleotides in a single chemical synthesis step (Patel *et al.,* 2015; Pressman *et al.,* 2015).

In addition to the generation of necessary prebiotic chemical precursors, there is growing recognition that minerals may have played a role in both the formation of simple organic molecules such as formaldehyde and, possibly, in the formation of molecules as complex as RNA, through reactions mediated by minerals, in particular clays (Brack, 2006). Ferris (2006, 2005) showed that the clay mineral montmorillonite is able to catalyze a variety of organic reactions, in particular the formation of RNA, and Fraser *et al.* (2011) suggested that the interlayer space in clays leads to the formation both of RNA oligomers and the selection of left-handed amino acids. It has also been hypothesized that clays could act as initial templates for the earliest self-replicating molecules (Cairns-Smith, 1966; Ponnamperuma *et al.,* 1982; Brack, 2006). Importantly, clays are ubiquitous products of meteorite impact events on Earth, being generated during the hydrothermal phase (Naumov, 2005; Osinski *et al.,* 2013) (see Section 2.2), an origin that has also been proposed to account for at least some of the clay detections on Mars (*e.g.,* Newsom, 1980; Schwenzer and Kring, 2009; Tornabene *et al.,* 2013).

In an intriguing set of experiments, McCaffrey *et al.* (2014) mixed the sugar glycolaldehyde with montmorillonite and subjected this mixture to shock pressures up to ∼25 GPa. Not only did a significant proportion of both glycolaldehyde and montmorillonite survive (*cf.* the discussion above in Section 3.1) but new biologically relevant molecules, including threose, erythrose, and ethylene glycol, were formed.

A synthetic view of the conditions at the site of impact craters leads to a number of conditions that could be conducive to an origin of life. Cockell (2006) suggested that because of the indiscriminate location of impact events in all lithologies and events of different sizes, leading to hydrothermal systems of different longevities and characterized by different geochemistry, impact craters offer the possibility of a large number of “experiments” in the origin of life, in comparison to, for example, volcanic hydrothermal systems. Specifically, conditions that might be conducive to origin-of-life scenarios are (1) production of diverse clays, zeolites, and mobilization of sulfides and other mineral catalytic and concentrating surfaces; (2) generation of vast mineral surface areas from impact fracturing and metamorphism that aid in catalysis and polymerization; (3) alkaline pH conditions; and (4) differential cooling regimes that in general move from high-temperature systems conducive to organic syntheses to cooler regimes conducive to complexification (Cockell, 2006).

In addition to clay minerals as substrates, shocked crystalline rocks may also have provided a form of concentration mechanism for the origins of life (Cockell, 2004). As described above, such rocks, when exposed to very high pressures, become highly porous and have a high surface area to volume ratio and can have densities lower than that of water, thus being similar to pumice—which can float and form aggregate rafts of material. Brasier *et al.* (2011) argued that such pumice rafts might have had a role in the origins of life. Here we suggest that the glassy material that comprises highly shocked rocks (similar to pumice) weathers into clays that can then adsorb metals, organics, and phosphates, providing both a reactive surface for prebiotic chemistry as well as an effective method for concentration of putative prebiotic molecules.

## 4. The Role of Impacts in Generating Habitats for Life

Notwithstanding the destructive effects of meteorite impact events as evidenced by the Cretaceous–Paleogene mass extinction event (Schulte *et al.,* 2010), it is now clear that impact events generate several habitats that are highly conducive to microbial colonization. These habitats increase planetary habitability; however, whether these habitats become inhabited depends on whether the ingredients and conditions for life are present in the near surface of the target. The key argument is that the formation of a meteorite impact crater results in the production of a habitat (or habitats) that was not present before the impact but that then can be viewed as being beneficial from a biological standpoint. The three major habitats generated by impact events are impact-generated hydrothermal systems, lithophytic habitats (both near surface and deep subsurface), and impact crater lakes, which are outlined and reviewed below.

### 4.1. Hydrothermal habitats

**FIG. 6.**
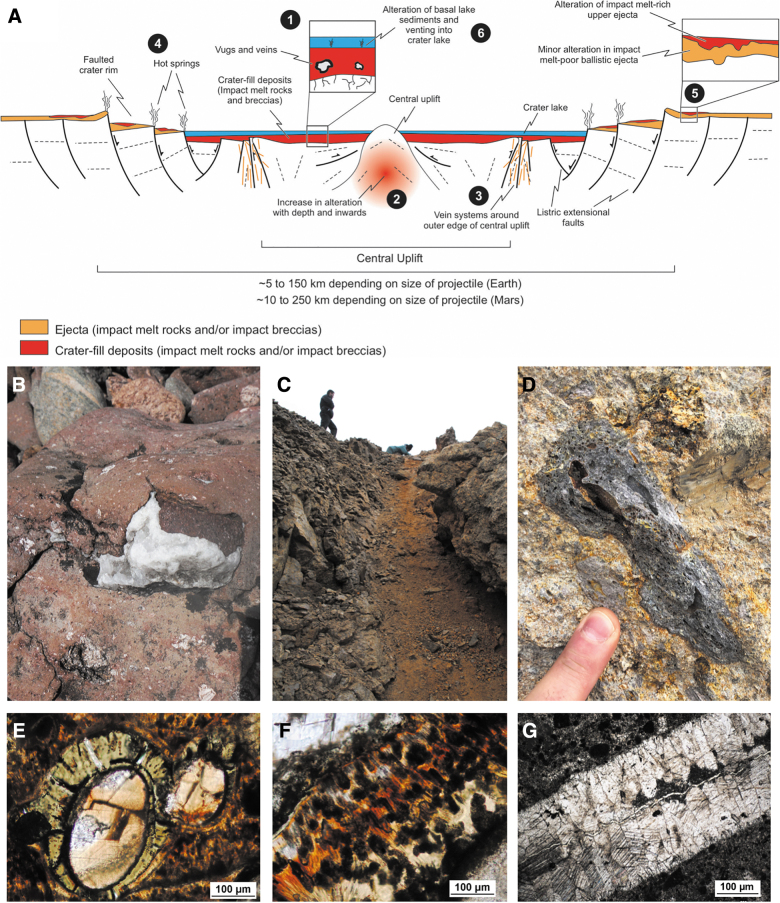
(**A**) Distribution of the six major settings for impact-generated hydrothermal alteration deposits within and around a typical complex impact crater (modified from Osinski *et al.,* 2013). (**B**) Quartz vug in crater-fill impact melt rocks, West Clearwater Lake impact structure, Canada. (**C**) Hydrothermal pipe structure interpreted as fossil hydrothermal vent in the rim region of the Haughton impact structure. (**D**) Altered impact melt-bearing breccia from the Ries impact structure, Germany. (**E**) Clay lining a vesicle within impact melt-bearing breccia from the Chicxulub impact structure, Mexico. (**F**) Zeolite within impact melt-bearing breccia from the Chicxulub impact structure, Mexico. (**G**) Calcite vein in the central uplift of the Haughton impact structure, Canada.

Hydrothermal systems in general have been widely proposed as a candidate habitat or “cradle” for the origin and early evolution of life on Earth (*e.g.,* Baross and Hoffman, 1985; Martin *et al.,* 2008; Russell *et al.,* 2013; Damer and Deamer, 2015, 2020; Deamer *et al.,* 2019) and possibly other planets such as Mars (*e.g.,* Farmer 2000; Schwenzer and Kring, 2009). In addition to being the potential environment in which life began, as discussed above, hydrothermal environments represent habitats that have likely played an important role in planetary habitability throughout geological time (Farmer, 2000). The origin and development of impact-generated hydrothermal systems have been presented in Section 2.3. As was noted, the habitable zones for hyperthermophilic and thermophilic organisms change significantly in space and volume during the evolution of an impact-generated hydrothermal system. Crater size plays an important role in determining the longevity of the hydrothermal system and in the spatial distribution of hydrothermal alteration (Kirsimäe and Osinski, 2012). Based on studies of several terrestrial impact structures, Osinski *et al.* (2013) distinguished six distinct locations in a typical complex impact crater where impact-generated hydrothermal deposits and habitats can form (Fig. 6A): (1) crater-fill impact melt rocks and melt-bearing breccias; (2) interior of central uplifts; (3) outer margin of central uplifts; (4) crater rim region; (5) impact ejecta deposits; and (6) post-impact crater lake sediments.

Impact melt-bearing impactites within the crater interior are typically both the major heat source for hydrothermal activity and the focus for most alteration (setting #1; Fig. 6A, 6B). The recognition of alteration phases within such lithologies led to the initial concept of impact-generated hydrothermal systems (*e.g.,* Newsom, 1980). Mineralization within crater-fill impact melt rocks and melt-bearing breccias ranges from discrete cavity (Fig. 6B) and fracture fillings (*i.e.,* vugs and veins) to being completely pervasive. The difference in intensity of hydrothermal alteration of crater-fill impactites has been ascribed to the presence/absence of an overlying crater lake (Osinski *et al.,* 2013). In terms of central uplifts, there is evidence for an increase in temperature and intensity of alteration both with depth and toward the crater center (Naumov, 2002) (Figs. 5, 6). The outer margin of central uplifts is also typically highly fractured and faulted and, correspondingly, provides sites of preferential fluid flow and hydrothermal mineralization (Hode *et al.,* 2003; Osinski *et al.,* 2005a) (Figs. 5, 6). An interesting finding from the past decade is the discovery of a spring mound located upon the so-called inner crystalline ring of the Ries impact structure that coincides with the outer edge of the central uplift. This feature displays travertine and evidence for microbial filaments interpreted as forming in subaerial spring effluents at ∼60–70°C (Arp *et al.,* 2013).

The rims of complex impact craters are characterized by large kilometer-scale faults formed during crater collapse representing excellent fluid pathways (Figs. 5, 6A). Detailed field mapping of the rim region of the Haughton impact structure revealed the presence of dozens of hydrothermal “pipe” structures that are subvertical cylindrical structures ranging from ∼1 to ∼5 m across and are exposed in outcrops over lengths of up to 20 m (Fig. 6C) (Osinski *et al.,* 2001, 2005a). These pipe structures have been interpreted as fossil hydrothermal vents, whose surficial expressions were likely hot springs and/or fumaroles.

In terms of volume, crater-fill deposits and central uplifts are the principal hydrothermal habitats; however, in terms of spatial extent, impact ejecta deposits dominate, and they represent an important hydrothermal habitat (Figs. 5, 6A, 6D). Indeed, impact ejecta deposits are one of the characteristic features of impact craters throughout the Solar System (*e.g.,* Osinski *et al.,* 2011), with continuous ejecta blankets extending from ∼1 to 3 crater radii, discontinuous deposits extending to ∼5 crater radii, and then the potential for distal ejecta deposits to be distributed over thousands of kilometers. As well as excavating preexisting hydrothermally altered rocks, new hydrothermal habitats can be generated in ejecta deposits (Figs. 5, 6D). The best-studied example of impact-generated hydrothermal alteration of ejecta deposits is at the Ries impact structure, Germany. At this site, there is little to no alteration of the continuous ejecta blanket (Fig. 6A) but abundant alteration of the overlying impact melt-bearing breccias (Newsom *et al.,* 1986; Osinski, 2005; Arp *et al.,* 2013; Sapers *et al.,* 2017) (Fig. 6D). Clays and zeolites are the dominant alteration assemblages, and it is important to note that the heat source for the alteration of ejecta deposits came entirely from the deposits themselves.

The final setting where impact-generated hydrothermal alteration has been proposed is within crater lake sediments (Figs. 5, 6A), which by themselves are important habitats (see Section 4.3). Impact crater lake sediments are relatively poorly studied, but the presence of clays and zeolites in the basal intra-crater lacustrine sediments at the Ries (Salger, 1977; Osinski, 2005) and Boltysh (Jolley *et al.,* 2010) structures is suggestive of hydrothermal alteration. A detailed study of the basal Ries crater lake sediments is ongoing and confirms the hydrothermal origin of smectite clays but also highlights that there is a complex story of other secondary mineral formation through recent weathering (Svensson *et al.,* 2019). Obviously, the alteration of these post-impact sediments implies that lake generation, sediment deposition, and hydrothermal activity all occurred congruently. The venting of hydrothermal systems into overlying lake environments could provide ideal habitats for life. This is particularly relevant for Mars, given both the ongoing Mars Science Laboratory and the Mars 2020 missions are focused on the investigation of intra-crater sedimentary deposits. We return to this in Section 6.

A wide range of hydrothermal minerals have been documented in impact structures around the world, with target lithology strongly influencing the mineral assemblages formed in impact-generated hydrothermal systems. Osinski *et al.* (2013) reported a detailed listing of primary minerals documented in terrestrial impact structures, with the dominant types being silicates (predominantly quartz [Fig. 6B], K-feldspar, clays [Fig. 6E], zeolites [Fig. 6F]), carbonates (*e.g.,* calcite [Fig. 6G]), sulfides, sulfates, halides, and various oxides and oxyhydroxides (Figs. 6C, 6D). A further study conducted at the Haughton impact structure, by Izawa *et al.* (2011), characterized the recent and ongoing weathering of primary minerals from the impact-generated hydrothermal system (Fig 2). These authors documented the alteration, remobilization, and reprecipitation of hydrothermal sulfide minerals in transient near-surface aqueous environments and oxidative near-surface conditions that produced a series of hydrated Fe-bearing oxides and sulfates (*e.g.,* fibroferrite, jarosite, copiapite). The authors outlined a series of metabolic pathways whereby chemolithotrophic microorganisms could have been involved in the weathering of hydrothermal sulfides. Sulfides are ubiquitous in impact-generated hydrothermal systems (Naumov, 2005) and provide an excellent source of reduced sulfur for sulfur-oxidizing chemotrophic metabolisms.

Is there evidence that these impact-generated hydrothermal systems were colonized by microorganisms? The most conclusive evidence thus far is sulfur isotope data from the Haughton (Parnell *et al.,* 2010b) and Rochechouart (Simpson *et al.,* 2017) impact structures that demonstrated extreme sulfur isotopic fractionation in hydrothermal sulfides relative to original sulfate lithologies, consistent with microbial sulfate reduction by thermophiles. Other more ambiguous evidence comes in the form of potential microfossils (Glamoclija, 2007; Lindgren *et al.,* 2010; Sapers *et al.,* 2015), microbial etching features (Glamoclija *et al.,* 2007), and fossilized extracellular polymeric substances (Hode *et al.,* 2008).

### 4.2. Near-surface lithophytic habitats

The ability of microorganisms to grow within rocks has long been recognized as an advantageous trait in harsh environments such as hot and cold deserts, particularly in surface environments where photosynthesis is possible (*e.g.,* Friedmann, 1982; Bell, 1993). This includes cryptoendolithic communities that live within the rock interstices and chasmoendolithic communities that live within rock fractures directly connected to the surface. Such habitats are able to provide protection against stressors such as large temperature shifts, low water availability, and high levels of UV radiation (*e.g.,* Cockell *et al.,* 2002; Walker and Pace, 2007; Omelon, 2008). Endolithic habitats are of interest in astrobiology, as they represent environments where physical conditions within rocks may be ameliorated in comparison to macroscale conditions (*e.g.,* Wynn-Williams and Edwards, 2000; Davila and Schulze-Makuch, 2016).

**FIG. 7.**
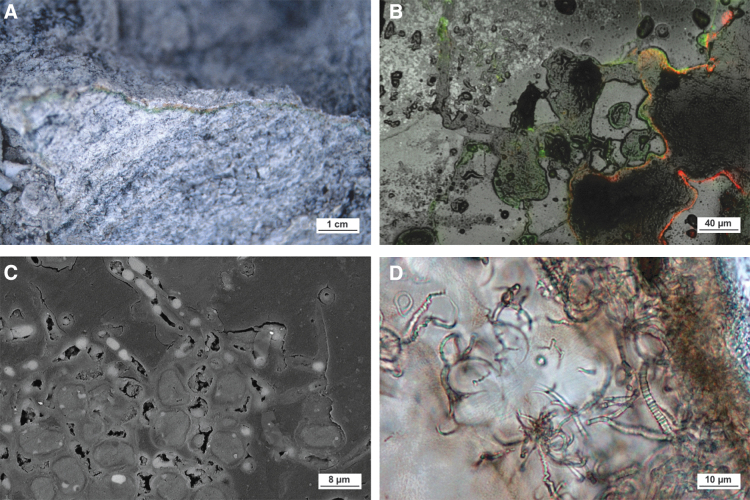
Endolithic lithophytic habitats in impactite lithologies. (**A**) Increased porosity in a shocked gneiss from the Haughton impact structure provides a physical habitat for a continuous band of cyanobacteria ∼2–5 mm below the rock surface (Cockell *et al.,* 2002). (**B**) Confocal laser scanning micrograph showing live (green) and dead (red) microorganisms colonizing void spaces in a shocked gneiss from the Haughton impact structure (modified from Pontefract *et al.,* 2014). (**C**) Backscattered electron microscope image of osmium-stained cyanobacteria and thylakoid membranes (white) colonizing a shocked gneiss from the Haughton structure. (**D**) Impact glass from the Ries impact structure provides a substrate for euendoliths (modified from Sapers *et al.,* 2015).

In non-impact settings, surficial endolithic habitats can be divided into microbial communities that are dependent on photosynthesis and its organic by-products and those completely decoupled from organic photosynthate. The former are typically restricted to sedimentary rocks, primarily sandstone, evaporites, and some carbonates, where the translucence, porosity, and permeability, are sufficient to allow for the penetration of photosynthetically active radiation (Friedmann, 1982). As described in Section 2.1, during the thermobaric phase of impact cratering, significant changes occur to the available surface area of the substrate, both through large-scale brecciation and faulting within the crater (Fig. 4) and through microscale changes in the porosity of the rocks (Fig. 3). Impact fracturing increases the fracture space within rocks, providing a greater abundance of habitats for chasmoendoliths in surface and near-surface impact crater environments (Cockell *et al.,* 2005). It has also been shown based on a series of studies on the Haughton impact structure that impact events also create new cryptoendolithic habitats (Fig. 7A–7C).

Surficial endolithic habitats are affected by the changes in porosity experienced by the target that are largely dependent on the structural and chemical composition of the rock. Sedimentary lithologies, which originally start porous, actually experience a loss of porosity and recrystallize at approximately 35 GPa (Cockell and Osinski, 2007; Osinski, 2007), while crystalline lithologies experience an increase in porosity with increased pressure (Figs. 3, 7A–7C) until melting at pressures exceeding ∼60 GPa (*e.g.,* Singleton *et al.,* 2011). In parallel, light transmission through the substrate has also been documented to increase, where penetration of photosynthetically active radiation can increase by an order of magnitude (Cockell *et al.,* 2002; Pontefract *et al.,* 2014). These shock-metamorphosed crystalline lithologies transform a previously uninhabitable substrate (generally on account of low porosity) into a moisture-retaining, UV-protected habitat for endoliths (Fig. 7A–7C), where the amount of biomass directly correlates with the level of shock metamorphism (Cockell *et al.,* 2002; Fike *et al.,* 2003; Cockell, 2004; Pontefract *et al.,* 2014). These impact-processed rocks contain a diverse set of nutrients essential to microorganisms, many of which are mobilized within glasses and thus can be accessed through the use of organic acids (Pontefract *et al.,* 2014). Moreover, these endolithic environments serve as nutrient traps in these limiting regimes, evidenced by the fact that shocked crystalline lithologies were dominated—especially at lower shock levels—by heterotrophic bacteria, which must have been reliant on an exogenous form of carbon in addition to that provided by primary producers (Pontefract *et al.,* 2016). In summary, through shock metamorphism, an impact event can transform the interior of a previously uncolonizable, dense, nonporous crystalline rock into a unique habitat for endolithic microorganisms both at the surface and at depth (see the next section).

The extreme end-member of impact processing is the melting of rocks during the thermobaric phase (Section 2.1). Rapid cooling or “quenching” of silicate impact melt results in the production of glass (see Osinski *et al.,* 2018, and references therein) that can be a highly beneficial substrate for microorganisms. It is worth noting that impact glasses are a ubiquitous product of impact events on Earth, being documented in craters from a few tens of meters in diameter (*e.g.,* the 45 m diameter Kamil Crater, Egypt; Folco *et al.,* 2011) to hundreds of kilometers across (*e.g.,* the 250 km diameter Sudbury impact structure; Dressler *et al.,* 1996). Impact glasses can be deposited as individual fragments in proximal and distal impact ejecta—including tektites and spherules that can be distributed globally (Glass, 1990; Simonson and Glass, 2004; Osinski *et al.,* 2018)—and as clasts in breccias, both in crater-fill deposits and in ejecta blankets (Figs. 2, 4). Long predicted to occur, impact glasses have also been identified on Mars and are thought to be widespread (Cannon and Mustard, 2015).

Impact glasses share many similarities with volcanic glasses, such as being translucent (when fresh and unaltered) and often containing quench crystallites of minerals such as pyroxene and feldspar (Stöffler, 1984). However, the bulk compositions of impact melts are diverse, reflecting heterogeneities in the target lithologies, and often display greater chemical heterogeneity on multiple scales than observed in comparative volcanic products (see Osinski *et al.,* 2018, and references therein). In addition to acting as a substrate for life as described in Section 3.2, glasses in general have a higher dissolution rate and, consequently, a higher availability of bioessential nutrients, thus providing an excellent habitat for potential microbial colonization (*e.g.,* Banerjee and Muehlenbachs, 2003; Cockell *et al.,* 2009b; Izawa *et al.,* 2010). Bioalteration of terrestrial basaltic glasses has been shown to produce characteristic tubular and granular aggregate textures (Furnes *et al.,* 2001). Such bioalteration textures preserved in Archean greenstone belts constitute one of the oldest records of life on Earth (Furnes *et al.,* 2004). Examination of glasses from the Ries impact structure in Germany has revealed tubular textures with remarkably similar morphologies (Fig. 7D), inconsistent with known mineralogical crystallization mechanisms. In addition to these morphological traits, evidence for organic carbon and unique iron speciation patterns lead to the conclusion that, as with their counterparts in volcanic glasses, these tubular structures in impact glasses represent microbial trace fossils (Fig. 7D) (Sapers *et al.,* 2014, 2015).

Given the high-temperature origin of impact glasses, it would seem unlikely that any biomarkers would be preserved, but this is exactly what has been reported in two studies. In the first, Howard *et al.* (2013) proposed that that biomarkers representative of pre-impact plant species are preserved in the “Darwin Glass,” found over an area of ∼400 km^2^ in Tasmania. Schultz *et al.* (2014) reported similar findings from purported impact glasses from Argentina.

A final notable aspect of impact melting is that on planetary bodies with substantial amounts of ices in their subsurface, impact melt would take the form of water, hydrocarbons, and so on. An excellent example of this is Saturn's moon Titan, where the average surface temperatures are too low (∼ -180°C) for liquid water to exist. However, as discussed by Neish *et al.* (2018), transient liquid water environments may be created by impacts in the form of impact melt deposits and, furthermore, subsequent reactions between organic molecules known to exist on Titan and these water-rich impact melts could produce a range of biomolecules such as amino acids. These authors conclude that the best sites to identify biological molecules on Titan are deposits of impact melt on the floors of large, fresh impact craters.

### 4.3. Deep subsurface habitats

In recent years, there has been a growing recognition of the importance of the deep subsurface biosphere on Earth (*e.g.,* Gold, 1992; D'Hondt *et al.,* 2002; Parkes *et al.,* 2011; Lollar *et al.,* 2014; Onstott *et al.,* 2019). While previously thought to be either minor components of the terrestrial microbial biomass or simply dying transplanted surficial communities, the subsurface biosphere is now estimated to account for 5–15% of all biomass on Earth comprising 27–64 Gt of carbon and hosting over 90% of all bacteria and archaeal biomass on Earth (Bar-On *et al.,* 2018; Magnabosco *et al.,* 2018). Data from these studies derives from drill cores and from deep underground mines primarily in the shield areas of Canada, South Africa, and Scandinavia in rocks varying from 2 to 3 Ga. These regions represent the ancient highly metamorphosed cores of continents and, while important on Earth, would not have been formed and exhumed if it were not for plate tectonics, which, to our knowledge, is lacking on all other objects in the Solar System at the present day.

In the previous section we showed that fracturing, shock metamorphism, and melting during the thermobaric phase can create new surficial endolithic habitats. That meteorite impacts result in faulting and fracturing to kilometers' depth is well established, both through field observations (*e.g.,* Kenkmann *et al.,* 2014) and gravity data (*e.g.,* Pilkington and Grieve, 1992). Seismic studies of various craters on Earth demonstrate that the faults, particularly in the rims of complex impact craters, can penetrate several kilometers, with the maximum fault depth scaling approximately with the size of the crater (*e.g.,* Pilkington and Grieve, 1992). However, it was not until the Gravity Recovery and Interior Laboratory (GRAIL) mission returned new gravity maps of the Moon that it was realized how important impact fracturing and faulting is on a planetary scale. GRAIL data revealed that the average porosity of the lunar crust is ∼12% and that this high porosity extends to depths of at least 10–25 km and possibly down to the mantle (Wieczorek *et al.,* 2013). Soderblom *et al.* (2015) further demonstrated that impact-generated fracturing is likely responsible for this high porosity.

Impact cratering thus provides a mechanism to fracture and fault planetary crusts down to several kilometers' depth. Perhaps as important is that these faults are connected to the surface, providing a pathway to connect the previously described hydrothermal, lithophytic, and crater lake habitats with the deep subsurface. Indeed, the vast majority of microorganisms in the subsurface are substrate-attached outnumbering pelagic or free-living cells by 1–3 orders of magnitude (McMahon and Parnell, 2014). The significance of substrate attachment underscores the importance of rock surface area and physicochemical characteristics highlighting the role that both impact-induced fracturing and shock metamorphism play in creating subsurface habitats.

Is there any evidence that impact events have influenced the deep subsurface biosphere on Earth? Investigations on the microbiology of a 1.76 km drill core obtained from the Chesapeake Bay impact structure in the United States (Gohn *et al.,* 2008), with robust contamination control (Cockell *et al.,* 2009a, 2012b; Gronstal *et al.,* 2009; Sanford *et al.,* 2009), showed a logarithmic downward decline in cell abundance consistent with the general trend of decreasing biomass with increasing depth. However, cell enumerations within the impact breccia revealed much more abundant biomass levels than would have been predicted based on the general trend indicated by the post-impact lithologies. When compared to previously studied subsurface environments, these communities are found to be consistent with a microbiota influenced by the diverse and mixed lithologies present in the impact melt-bearing breccias. Coupled with the low hydraulic conductivity, the data suggest the microbial community remains influenced by the impact ∼35 million years ago (Cockell *et al.,* 2009a). While the discovery of relatively high biomass in impactite units at depth is intriguing, detailed metagenomic and biogeochemical studies are required to model the energy regimes and metabolic potential of subsurface impact environments. These data show that although impacts will sterilize the immediate area during the event itself, the fracturing caused by impact can yield enhanced habitat for microorganisms over the long term.

### 4.4. Lacustrine habitats

Lacustrine sediments in general offer not only a quiescent habitat allowing for the establishment of stable microbial communities but also excellent preservation potential of organics and other biomarkers (*e.g.,* Meyers and Ishiwatari, 1993). Lakes in themselves are not environments unique to impact craters; however, what makes impact crater lakes unique is that craters represent anomalous topographic basins that can form anywhere on a planet and on any planetary object where the conditions permit the existence of water (or other fluid such as hydrocarbons on Titan). Indeed, even on Earth, many impact craters have been targeted for scientific studies not because they are craters but because of the paleoclimate history recorded in their crater lake sediments, a history that is not preserved in surrounding regions, largely because lakes of similar depth and/or longevity do not exist. Notable examples include the Bosumtwi (see Koeberl *et al.,* 2007, and references therein) and El'gygytgyn (see Koeberl *et al.,* 2013, and references therein) impact structures that were drilled by the International Continental Scientific Drilling Program (ICDP). Both craters have provided unique paleoclimate records in West Africa and Arctic Russia, respectively. On other planetary bodies, where plate tectonics and volcanic activity are either lacking or restricted, impact craters provide one of the only ways in which large, deep sedimentary basins are formed. Indeed, on Mars, impact crater lake sediments have received considerable attention as astrobiological targets (*e.g.,* Grin and Cabrol, 1997; Cabrol and Grin, 1999, 2001), which we return to in Section 6.

**FIG. 8.**
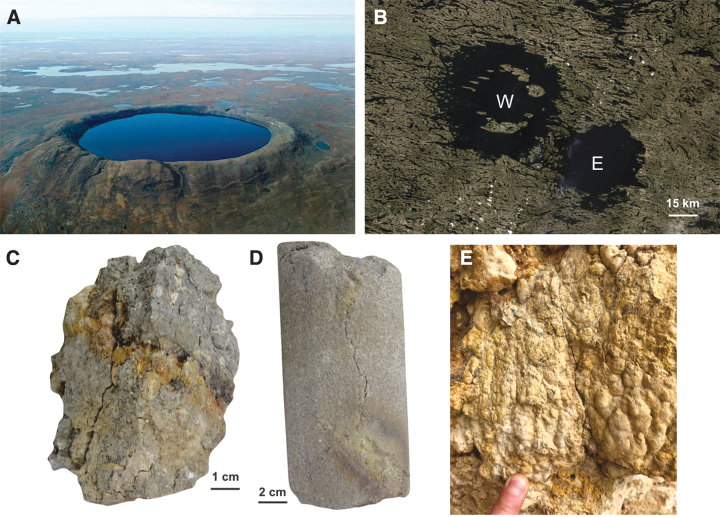
Impact crater lake environments and habitats. (**A**) The 3.44 km diameter New Quebec or Pinguluit Crater is a well-preserved simple impact crater in northern Quebec, Canada. The crater lake is ∼270 m deep and has no inlets or apparent outlets. (**B**) The West (“W”) and East (“E”) Clearwater Lake impact structures in Quebec are ∼36 and ∼26 km in diameter, respectively, and both contain crater lakes. (**C**) Hydrothermally altered coarse-grained sediments deposited at the base of the Ries crater lake. The orange color is due to hydrothermal iron oxides and clays. Sample is from 14.3 m depth in the Wörnitzostheim drill core. (**D**) Alteration halo around a calcite-lined cavity in fine-grained lake sediments in the Nördlingen drill core (323 m depth), Ries impact structure. (**E**) Stromatolitic limestone from the upper part of the Ries crater lake succession. Finger for scale.

As described in Section 2.2 and shown schematically in Fig. 5, in the weeks and months following the impact event, once the conditions are appropriate, the combined influx of groundwater, precipitation, seawater, and/or water from the melting of surface and subsurface ice deposits, or liberated from mineral structures due to shock metamorphism and heating, can lead to the generation of a crater lake. Once the area of the impact has begun to cool, opportunities for the synthesis of prebiotic molecules and compounds increase. The continuance and conservation of these chemical reactions is largely dependent on whether a crater lake forms within the impact basin. Examination of impact crater lakes on Earth shows that the underlying morphological conditions and chemistry result in significant variations in present-day biology. Some craters have steep walls and consequently more oligotrophic lake profiles due to shallow littoral regions (*e.g.,* in the New Quebec Crater [Fig. 8A]; Cockell and Bland, 2005). Alternatively, others such as the West Clearwater Lake structure in Quebec host a lake with an internal island ring (Fig. 8B). This lake has a richer periphyton community (*i.e.,* organisms that live attached to underwater surfaces) than New Quebec likely owing to the increased area for littoral zone organisms (Cockell and Lee, 2002). By contrast, the Tswaing impact crater lake that formed in a 1.13 km diameter simple crater is an evaporitic lake containing a rich photosynthetic microbial community (Schoeman and Ashton, 1983; Ashton and Schoeman, 1988). Furthermore, the target substrate can also influence the microbial diversity of the impact lake: impact crater lakes forming in volcanic targets show a huge level of diversity in their biology (*e.g.,* Schoeman and Ashton, 1983; Gronlund *et al.,* 1990; Maltais and Vincent, 1997), determined by rock-water interactions that can influence salinity, temperature, and dissolved oxygen concentrations. These observations show that local geology and long-term geological processes can have profound influences on impact crater lake biota, generating great diversity in impact crater lake hosted communities (Cockell and Lee, 2002).

Water levels in these lakes change over time, either due to environmental changes or erosion of crater rims. When a lake is no longer present, these changes and any biology that once inhabited the crater can be well preserved in the lacustrine sediments that overlie crater-fill impact melt rocks and breccias (Fig. 8C–8E). The high organic content of the sediment can also give way to later ecological succession in the form of macroflora and macrofauna. As noted in Section 2.2, the Haughton impact structure is an excellent example of the preservation potential of crater lakes, where the stratigraphic record contained with the Haughton Formation (the crater lake sediments) represents the only record of the Miocene period in the Canadian Arctic (Hickey *et al.,* 1988; Osinski and Lee, 2005; Rybczynski *et al.,* 2009) and provides a present-day fertile substrate (Cockell *et al.,* 2001).

The Haughton example is one where the crater lake sediments preserved as the Haughton Formation date to the post-impact succession phase, as they were deposited following a period of a few million years of erosion (Osinski and Lee, 2005). It is unknown as to whether a crater lake was formed immediately following impact. At the Ries impact structure in Germany, a thick series of crater lake sediments (∼400 m) are preserved, and drill cores (Fig. 8C, 8D) and surface outcrops (Fig. 8E) afford unique insight into the development of crater lakes in general. Microbial mats are common in the upper successions (Fig. 8E). Importantly, as discussed above in Section 4.1, the basal few meters of these lake sediments are hydrothermally altered (Fig. 8C, 8D) (Osinski, 2005; Svensson *et al.,* 2019). This is a very understudied impact crater habitat, but we note that Chatterjee (2016) has proposed hydrothermal impact crater lake environments as possible environments suitable for the emergence of life, although that author's model of a magma chamber at depth driving hydrothermal systems within impact craters is not consistent with observations from craters on Earth as described herein.

## 5. Impact Craters as Cradles for the Origin of Life and Its Subsequent Survival

In Section 3, we reviewed how meteorite impacts could have delivered and generated the necessary ingredients for life—ranging from amino acids, HCN and CO, to clay minerals—on Hadean Earth. In Section 4, we discussed how impact events can also create unique new habitats for life. In this section, we return to the question of the environment (or environments) in which life originated on Earth. We draw extensively from the work of Westall *et al.* (2018), who provided a recent summary of the necessary steps for the transition from prebiotic reactions to biotic entities to have occurred and the myriad of environments where this could have taken place. In addition to proposing a new environment, in the form of the sedimentary layer between oceanic crust and seawater, Westall *et al.* (2018) categorized four main other potential environments for the origin of life based on the literature (Table 2). These authors also provided their view as to the advantages and disadvantages of these various settings with respect to prebiotic chemistry and the emergence of life and assessed potentials for these environments in terms of origination (*i.e.,* the ability of the environment to provide the building blocks for life), complexification (*i.e.,* the ability of the environment to provide and sustain the conditions suitable for emergence of life), and plausibility (*i.e.,* the likelihood that this environment existed on Hadean Earth). Westall *et al.* (2018) concluded that submarine hydrothermal vents—previously proposed by many in the literature (*e.g.,* Baross and Hoffman, 1985; Corliss, 1996; Russell and Hall, 1997; Martin *et al.,* 2008; Deamer and Georgiou, 2015)—and their own proposed hydrothermal-sedimentary ocean seafloor setting are the two most likely candidate environments for the origin of life (Table 2). This is consistent with the work of Russell *et al.* (2013), who proposed that cellular life was preceded by abiotic metabolisms derived from the transition of physical energy, resulting from seafloor fracturing, to chemical energy through the process of serpentinization during the formation of precipitated hydrothermal mounds. Russell *et al.* (2013) also argued that methane and hydrogen gasses released during serpentinization could have reacted with available electron acceptors (CO_2_, NO_3_, NO_2_, Fe^2+^, Mg^2+^) in the acidic Hadean ocean, satisfying the requirement for a -Δ*G*. Furthermore, the resulting pH gradient between the highly alkaline effluent and the acidic ocean could have driven the first primitive proton pumps across a hydrothermal mound. These initial processes would have allowed for increased complexity and the generation of pyrophosphate in ferrous hydroxide layers, acting as highly reactive and permeable “membranes,” allowing for concentration of prebiotic constituents. Although these hydrothermal vent and hydrothermal-sedimentary environments are set in the context of submarine volcanism, we contend that these processes could also have occurred in impact-generated hydrothermal environments on Hadean Earth.

**Table 2.** Proposed Environments for the Origin of Life on Earth Due to Endogenic Processes and Equivalent Impact-Generated Origin

Environment	Endogenic origin^*^	Impact-generated origin?
Submarine hydrothermal vents	Active at the present day along oceanic spreading ridges (black smokers) and along off-ridge spreading centers (white smokers) (*i.e.,* due to plate tectonics). Proposed to have formed in the Hadean due to higher heat flow in crust driving abundant volcanic activity.	Formed due to impact-generated hydrothermal activity in marine impact craters. Venting around faulted crater rims and central uplifts documented in the literature.
Subaerial hot springs and geysers	Subaerial hot springs and geysers found in various continental settings. Heat sources include plate tectonic-derived volcanism, mantle hotspots, and radioactive decay.	As above for impacts on land. Creation of subaerial hot springs also possible for marine impacts, where the impact process leads to uplift of crater rims and central uplift (central peak and peak rings).
Volcanic splash pools	Formation of rock pools on newly formed lava flows in coastal volcanic environments.	Formation of rock pools on newly formed impact melt flows in coastal marine and crater lake environments.
Hydrothermal-sedimentary context	Hydrothermal fluid flow through the sedimentary layer between oceanic crust and seawater. Hydrothermal fluids derived from crust from volcanic activity.	Hydrothermal fluid flow through intra-crater sediments overlying impact melt rocks and breccias. Driven by impact-generated hydrothermal system.
Pumice rafts	Highly vesicular volcanic glass formed silica-rich (felsic) magmas during explosive volcanism. Volcanism during Hadean largely basaltic with small amounts of felsic volcanism proposed.	Pumice-like material formed during impact events and emplaced within and around craters as clasts in breccias.

^*^ See Westall *et al.* (2018) and references therein.

As outlined in Sections 2.2 and 4.1, the creation of hydrothermal systems is expected to occur for all craters above a few kilometers in diameter on Earth. Such impact-generated hydrothermal systems are not restricted to craters formed in continental settings. Indeed, hydrothermal activity is associated with two of the three largest structures on Earth—namely Sudbury (*e.g.,* Ames *et al.,* 1998, 2004a) and Chicxulub (*e.g.,* Ames *et al.,* 2004b; Zürcher and Kring, 2004)—which both formed in a shallow marine setting. The generation of submarine hydrothermal vents is, thus, an expected outcome of meteorite impacts into marine settings (Table 2). Furthermore, as discussed in Section 2.1, a predicted outcome of the frequent large impact events on Hadean Earth is that ultramafic rocks would have been exhumed by impact events via the formation of central uplifts and impact ejecta deposits. Such rocks, instantaneously brought to the surface of Earth, would be out of equilibrium and expected to undergo rapid alteration, providing an alternative mechanism for serpentinization in addition to the classic plate tectonic models.

The generation of submarine hydrothermal vents through meteorite impacts also circumvents a major problem for this origin-of-life hypothesis; namely that it is very uncertain as to whether black and white smoker-type environments—which at the present-day are associated with mid-ocean ridges—would have existed on early Earth due to the purported absence of plate tectonics (*e.g.,* Kamber, 2015). Furthermore, while modern mid-ocean-ridge-hosted hydrothermal systems have vent temperatures too high (>400°C; Von Damm, 1990) to support biological activity inside the vents, numerical modelling of the impact-generated hydrothermal systems at the aforementioned Sudbury and Chicxulub marine impact structures show some regions of the structure are immediately at temperatures in the range of 50°C to 100°C, with fluid flow climbing to >20,000 km^3^ after only 100,000 years and higher volumes still at 1 million years (Abramov and Kring, 2004, 2007).

In terms of a possible hydrothermal-sedimentary context for the origin of life, Westall *et al.* (2018) considered this environment exclusively in the context of the sedimentary layer between oceanic crust and seawater (Table 2). We propose that two possible hydrothermal-sedimentary environments are plausible within impact craters. The first is akin to the scenario of Westall *et al.* (2018), whereby marine meteorite impacts on the scale of Chicxulub and Sudbury generate large volumes of impact melt rock (see Section 2.1), which for all intents and purposes is equivalent to the oceanic crust, and have been shown to have been rapidly infilled by sediment, and also generate hydrothermal systems (see Section 2.2). It is outside the scope of this contribution to compare and contrast in detail this impact-generated hydrothermal-sedimentary environment with a volcanic one, but we note that many of the advantages outlined by Westall *et al.* (2018)—such as ideal temperatures and pH for thermophilic organisms, ideal mineral substrates, and availability of organics—are translatable to the impact setting. As discussed above in Section 4.3 and shown in Fig. 5, meteorite impacts also generate deep crater lakes and concomitant hydrothermal activity, providing a second impact-generated hydrothermal-sedimentary environment in continental settings.

In addition to submarine hydrothermal vents, there has been increasing interest in the possibility that subaerial hot springs and geysers may have provided a suitable environment for life's origin (Table 2). Such springs have been shown to have suitable fluid chemistries and sources of minerals and organics, in addition to generally lower temperature vent environments than black smoker submarine vents (see Cawood and Pirajno, 2008, and references therein). Other factors, such as cycles of wetting and drying characteristic of hot springs on land, have been used by some to suggest that these terrestrial environments were more conducive for life than their submarine counterparts (*e.g.,* Deamer and Georgiou, 2015; Deamer *et al.,* 2019).

When reviewing the plausibility of the subaerial hot springs and geysers origin-of-life setting, Westall *et al.* (2018) cited a major issue, which is the surmised lack of continents in the Hadean (Kamber, 2015). Yet again, impact craters provide a mechanism to generate subaerial settings even in marine environments—albeit with an upper limit of a few hundred meters of seawater—in the form of uplifted crater rims (Figs. 1, 4, 5), central peaks (Fig. 1B), and peak rings (Fig. 1C) (Table 2).

While undoubtedly less common on early Earth than the previously discussed environments, pumice rafts (Brasier *et al.,* 2011) and volcanic-hosted splash pools (Fox and Strasdeit, 2013) have also been proposed as potential environments for the origin of life. The formation of “impact pumice” has previously been described in Section 2.1 and shown in Fig. 3F (see also Table 2). The idea behind volcanic-hosted splash pools is the creation of rock pools on freshly erupted lava flows on coastal environments on Hadean Earth. As reviewed recently by Osinski *et al.* (2018), the emplacement of flows and ponding of impact melt in topographic lows both within the crater interior and in the ejecta deposits of complex craters is commonplace (see Fig. 4). Whether it be marine impacts or continental impacts that rapidly form crater lakes, we suggest that impact analogues for volcanic-hosted splash pools would likely have formed (Table 2), although we acknowledge that they would have been volumetrically minor compared to previously described settings. Westall *et al.* (2018) suggested that the major drawback for these two settings was their inability to generate their own organic and prebiotic complement and their short-lived nature. We suggest that this would not pose a problem for impact-generated pumice and splash pools, given the availability of the building blocks for life in as described in Section 3.

In the end, we may never know what environment on Earth life originated in, but we propose that meteorite impact craters offer all the necessary ingredients, substrates, and environments suitable for the emergence of life, and do so at a high rate of incidence. Indeed, as discussed above, from submarine hydrothermal vents to pumice rafts, impact craters represent an underappreciated mechanism to generate the various proposed environments for the origin of life. As has been noted previously by Cockell (2006) and further demonstrated here, impact craters could be considered a literal application of Darwin's “warm little pond.” Craters offer the added benefit of potentially creating all of these environments in close proximity, essentially representing an “origins” diversity hot spot. Furthermore, due to the inherent dynamic nature of the impact cratering process, the key ingredients for life and even the habitats themselves would have been mixed and dispersed over large areas of Earth's surface. Finally, unlike plate tectonics—which has substantial implications for the nature of volcanism on early Earth—we know that impact events were ubiquitous during the Hadean, thus reducing the necessity to rely on speculation as to the geological processes active on early Earth. Even if life did not originate in an impact crater or with building blocks provided by impact events, we propose that impact craters would have provided protected niches where life may have survived and eventually thrived during the Hadean and into the early Archean.

## 6. Implications for the Search for Life on Mars

It is widely believed that Mars and Earth shared similar early histories before their geological evolution diverged. Mars possesses all the key ingredients for life as we know it (McKay, 1997). As such, the search for life on the Red Planet has largely been driven by the mantra “follow the water” (*e.g.,* Hubbard *et al.,* 2002) and, more recently, the search for habitable environments (*e.g.,* Grotzinger, 2009). Whether Mars was once warm and wet or has always been cold and dry remains debated in the scientific community. On the one side, there is geomorphological (*e.g.,* lakes, rivers, and even oceans; Carr, 1996) and chemical (*e.g.,* detection of clays and salt minerals; Gendrin *et al.,* 2005; Poulet *et al.,* 2005; Ehlmann *et al.,* 2011) evidence for the presence of water on the surface of Mars early in its history. Increasingly, however, evidence suggesting that Mars was always cold and repeatedly glaciated has been presented (*e.g.,* Fastook and Head, 2015; Cassanelli and Head, 2019). This hypothesis is more consistent with the faint young Sun hypothesis (Wordsworth, 2016), creating uncertainty around the habitability of early Mars.

Evidence for volcanism is widespread on Mars (*e.g.,* Greeley and Spudis, 1981; Carr, 2006) and certainly, if conditions were favorable, all the environments for life outlined in Table 2 could, in theory, have formed. A major unknown, however, is whether standing bodies of water existed on early Mars for any significant length of time; thus, the formation of submarine hydrothermal vents, volcanic splash pools, and a hydrothermal-sedimentary setting remains speculative. The formation of subaerial hydrothermal systems driven by endogenic activity seems entirely plausible (*e.g.,* Schulze-Makuch *et al.,* 2007).

In contrast, meteorite impact craters are one of the most dominant geological landforms on Mars (Strom *et al.,* 1992). Given the evidence for water on Mars—whether in its liquid or solid state—we contend that the impact-generated and/or delivered ingredients, substrates, and environments for life on Hadean Earth (see Table 2) would all have been present on early Mars.

In terms of the chemical ingredients for life, given the lower average impact velocities and thinner atmosphere compared to Earth, the delivery of intact organic molecules and volatiles to early Mars is expected. Indeed, building on the work of Chyba and Sagan (1992) for Earth, Frantseva *et al.* (2018) performed dynamical simulations, which suggest that the delivery of organics from asteroids and comets is not only viable on Mars but may dominate over IDPs. As discussed previously, clay minerals may have played a role in the formation of simple and complex organic molecules. Clays have been widely detected on the surface of Mars from orbit (*e.g.,* Poulet *et al.,* 2005; Mustard *et al.,* 2008; Carter *et al.,* 2010; Ehlmann *et al.,* 2011). The vast majority of these clay detections occurs either within impact craters or in the heavily cratered southern highlands of Mars. Most workers interpret this association to be the result of the impact excavation of pre-impact clay minerals. Alternatively, given the likelihood of impact-generated hydrothermal activity on Mars (Newsom, 1980; Brakenridge *et al.,* 1985; Rathbun and Squyres, 2002; Osinski *et al.,* 2013), other workers have suggested that many clay-bearing deposits may be impact-generated (Tornabene *et al.,* 2013). Indeed, evidence for impact-generated hydrothermal systems on Mars has been reported (Marzo *et al.,* 2010; Osinski *et al.,* 2013).

With respect to rocky habitats, the effects and products of hypervelocity impact on Mars will essentially be the same as on Earth. Thus, we predict that highly shocked and shock-melted rocks on Mars will—as we have discussed in Section 4.2—be present and act as habitats. Of note is that the detection of impact glasses from orbit has recently been accomplished (Cannon and Mustard, 2015; Cannon *et al.,* 2017). Impact crater lakes have also been proposed to form on Mars, and evidence for such features is widespread throughout the heavily cratered southern highlands of Mars (*e.g.,* Cabrol and Grin, 1999; Goudge *et al.,* 2015). It is notable that the Mars Science Laboratory Curiosity rover is currently exploring and documenting a thick succession of crater-fill sediments within Gale Crater and the upcoming NASA Perseverance rover mission will land in Jezero Crater to explore similar crater-fill sediments.

In summary, we hypothesize that impact craters would have provided conditions suitable for the emergence of life on Mars through the production of substrates for prebiotic chemistry and through the production of habitats for the emergence and subsequent survival of microbial life. We propose that martian impact craters should be viewed as prime astrobiological landing sites, not for the secondary sedimentary record they contain (primarily the case for Gale and Gusev) but for the primary potential impact-generated habitats and substrates that they could contain.

We now turn our attention to the question of whether life could exist on present-day Mars. The surface of Mars today is a cold, arid, and radiation-intense environment, with evidence for only limited and transient habitable surface environments (*e.g.,* gullies, subglacial environments). Exactly when the transition from a potentially habitable surface to an inhospitable one occurred is not known. However, in extreme locations on Earth, surface conditions render the interior and subsurface of rocks, and soils beneath them, as preferential refugia for the propagation of life. Regardless of either an early warm and wet or cold and dry Mars, by the time the earliest evidence for life on Earth was recorded in the rock record, due to loss of the magnetic field by ∼3.9–4.1 Ga (*e.g.,* Acuña *et al.,* 1999) and the subsequent significant loss of atmosphere by 3.7 Ga (*e.g.,* Wordsworth, 2016; Bristow *et al.,* 2017), the surface of Mars was largely inhospitable due to the ionizing radiation and instability of surface water by 3.5 Ga.

It can be argued that relatively stable climatic conditions and a widespread surficial ocean (Valley *et al.,* 2002) were required for life to gain a significant hold on early Earth to leave behind a record, and certainly the dominance of surficial life on Earth is due to the evolution of oxygenic photosynthesis at ∼2.5 Ga, conditions that arguably never occurred on Mars. As such, the largest and longest-lived putatively habitable environment on Mars is in the subsurface (*e.g.,* Boston *et al.,* 1992). Impact craters would provide not only transiently habitable surface conditions and protective endolithic environments from UV and cosmic radiation but also provide connectivity to the subsurface. If life ever did exist on Mars, the ideal place to seek potential biosignatures of these last remnants, therefore, would be lithic habitats (*cf.* Onstott *et al.* 2019). The interior of shocked lithologies and substrates formed in impact hydrothermal systems would be one such location.

As described previously, the impact process can result in catastrophic fracturing and brecciation of the terrestrial subsurface on the scale of kilometers. Although this process may result in sterilization of the deep subsurface near the point of impact, the subsequent generation of the hydrothermal system, and the eventual cooling and cessation of that system, would leave behind fracture networks that could be exploited by microorganisms (see Section 4.4). In many subsurface environments, life is generally not limited by space but by energy and nutrients (*e.g.,* Hoehler and Jørgensen, 2013). Thus, impact-induced fracturing is suggested to improve fluid flow and thus access to nutrients and energy as is observed at Chesapeake (Cockell *et al.,* 2009a). Combining impact-brecciated rocks with the deep fractures formed during the impact, it is plausible that these subsurface martian rocks could theoretically have provided refugia for organisms as they do on Earth. In this way, impact cratering could enhance fluid flow and thus habitability of the deep subsurface on Mars (Cockell *et al.,* 2012a), preserving habitable conditions long after the surface conditions became largely inhospitable.

Notwithstanding the general inhospitable nature of the martian surface, it should be noted that impact events into the midlatitudes of present-day Mars—which contain vast amounts of water ice in the upper few 100 m—could also generate hydrothermal systems through the melting of ground ice and the subsequent development of transient crater lakes (Barnhart *et al.,* 2010; Ivanov and Pierazzo, 2011; Osinski *et al.,* 2013), two important habitats discussed in Section 4. It is not possible to accurately date individual craters on Mars, but based on knowledge of present-day impact rates, craters in the tens of kilometers' size range are predicted to have formed during the past 50 million years. An excellent example is the 27 km diameter Tooting Crater, which may have formed as recently as 3 Ma (Mouginis-Mark and Boyce, 2012). As discussed in Section 2.2, craters of this size range are expected to produce hydrothermal systems with durations of a few tens to hundreds of thousands of years. If sufficient ice was present in the subsurface, it seems entirely plausible that transient impact crater lakes would have formed. Their upper reaches would have rapidly frozen, but hydrothermal venting may have kept their lower depths liquid for a substantial period of time—essentially for the duration of the impact-generated hydrothermal system. An important consideration for current and planned life-detection missions in light of the unlikeness of a surficial biosphere is the role such impacts play in putative habitat connectivity. With the subsurface the most likely habitat for extant life on Mars, such impact events would have provided a physical connection between a putatively inhabited subsurface and a potentially habitable surficial environment.

## 7. Concluding Remarks

Impact events are a ubiquitous planetary geological process, and indeed, historically, their effects on planetary surfaces and subsurfaces have been largely examined through the lens of geology. As reviewed here, there is a growing realization that impacts can influence the biological evolution of planetary bodies. From the delivery of the building blocks for life, to the generation of substrates suitable for prebiotic chemistry, to the creation of a wide range of habitats for early life, we propose that meteorite impact events can significantly enhance habitable conditions on, and within, planetary bodies, inviting a literal application of Darwin's “warm little pond” hypothesis for the origin of life (*cf.* Cockell, 2006). Impact craters can provide all the previously proposed environments for the origin of life on Earth: subaerial and submarine hydrothermal vents, hydrothermal–sedimentary settings, and impact analogues for volcanic pumice rafts and splash pools. Rather than regard impact events as ephemeral exogenous and destructive components of planetary evolution, our hope is that this review demonstrates that we must regard meteorite impact events as a critical factor when considering the emergence and development of habitable conditions on planetary bodies.

Looking beyond Earth, as we have discussed in this review, we suggest that impact craters should be considered as prime sites in the search for evidence of past life on Mars and may have increased the habitability of the subsurface, with implications for the potential of extant life on Mars. While not within the scope of this review, we contend that impacts could increase the potential for any planetary body with the necessary ingredients for life—which could also be impact-delivered—to be at least transiently habitable. From large asteroids, such as Ceres, to satellites of the outer Solar System (*e.g.,* Titan), to exoplanets, we suggest that the concept of habitable zones and what factors influence planetary habitability should be reconsidered with meteorite impacts in mind.

In closing, given the incomplete nature of the rock record on Hadean and Archaean Earth, we may never know how or where life started on this planet. However, we speculate that, given the ubiquitous nature of impact events and their increased frequency during the first half billion years of Solar System history, meteorite impact craters may represent the most likely site (or sites) where life originated on Earth. This is a hypothesis that could eventually be tested on Mars or other planetary bodies. Would it not be poetic that impacts, long seen as harbingers of death, turn out to have in fact been the cradle of life?

## Notes

Associate Editor: Lewis Dartnell

## Abbreviations Used

D: crater diameter
IDPs: interplanetary dust particles
LHB: Late Heavy Bombardment
LUCA: last universal common ancestor
SU: structural uplift
